# Dysfunction of homeostatic control of dopamine by astrocytes in the developing prefrontal cortex leads to cognitive impairments

**DOI:** 10.1038/s41380-018-0226-y

**Published:** 2018-08-20

**Authors:** Francesco Petrelli, Glenn Dallérac, Luca Pucci, Corrado Calì, Tamara Zehnder, Sébastien Sultan, Salvatore Lecca, Andrea Chicca, Andrei Ivanov, Cédric S. Asensio, Vidar Gundersen, Nicolas Toni, Graham William Knott, Fulvio Magara, Jürg Gertsch, Frank Kirchhoff, Nicole Déglon, Bruno Giros, Robert H. Edwards, Jean-Pierre Mothet, Paola Bezzi

**Affiliations:** 1 Department of Fundamental Neurosciences, University of Lausanne, CH-1005 Lausanne, Switzerland; 2 Centre de Recherche en Neurobiologie et Neurophysiologie de Marseille, Aix-Marseille Université UMR7286 CNRS, 13344 Marseille, Cedex 15 France; 3 BESE division, King Abdullah University of Science and Technology, 23955-69000 Thuwal, Saudi Arabia; 4 Institute of Biochemistry and Molecular Medicine (IBMM), University of Bern, Buehlstrasse, 28 3012 Bern, Switzerland; 5 “Biophotonics and Synapse Physiopathology” Team, UMR9188 CNRS – ENS Paris Saclay, 91405 Orsay, France; 6 Departments of Neurology and Physiology, University of California San Francisco, San Francisco, CA 94158 USA; 7 CMBN, Rikshospitalet, University of Oslo, Oslo, Norway; 8 BioEM Facility, School of Life Sciences, Ecole Polytechnique Fédérale de Lausanne, CH-1015 Lausanne, Switzerland; 9 Centre for Psychiatric Neuroscience, Department of Psychiatry, Lausanne University Hospital Center, University of Lausanne, CH-1015 Lausanne, Switzerland; 10 Department of Molecular Physiology, University of Saarland, D-66421 Homburg, Germany; 11 Department of Clinical Neurosciences, Lausanne University Hospital, Lausanne, Switzerland; 12 Neuroscience Research Center, Lausanne University Hospital, CH-1011 Lausanne, Switzerland; 13 Department of Psychiatry, Douglas Mental Health University Institute, McGill University, Montreal, Quebec H4H1R3 Canada; 14 INSERM, UMRS 1130; CNRS, UMR 8246; Sorbonne University UPMC, Neuroscience Paris-Seine, F-75005 Paris, France

**Keywords:** Neuroscience, Physiology

## Abstract

Astrocytes orchestrate neural development by powerfully coordinating synapse formation and function and, as such, may be critically involved in the pathogenesis of neurodevelopmental abnormalities and cognitive deficits commonly observed in psychiatric disorders. Here, we report the identification of a subset of cortical astrocytes that are competent for regulating dopamine (DA) homeostasis during postnatal development of the prefrontal cortex (PFC), allowing for optimal DA-mediated maturation of excitatory circuits. Such control of DA homeostasis occurs through the coordinated activity of astroglial vesicular monoamine transporter 2 (VMAT2) together with organic cation transporter 3 and monoamine oxidase type B, two key proteins for DA uptake and metabolism. Conditional deletion of VMAT2 in astrocytes postnatally produces loss of PFC DA homeostasis, leading to defective synaptic transmission and plasticity as well as impaired executive functions. Our findings show a novel role for PFC astrocytes in the DA modulation of cognitive performances with relevance to psychiatric disorders.

## Introduction

Acquisition of higher cognitive functions (i.e., executive functions) depends on the proper development and maturation of the prefrontal cortex (PFC) in both humans and rodents [1]. In humans, executive functions develop throughout childhood and adolescence, and the appropriate maturation of the circuitry within PFC may play a key role in this trajectory [2]. Consistent with this notion, impairments in executive functions are central symptoms associated with developmental neuropsychiatric disorders such as schizophrenia and autism spectrum disorders [1], thus suggesting that alterations in the development of synaptic circuitries in the PFC play a central role in the pathophysiology of psychiatric disorders with shared deficits in executive functions. This conceptual framing of psychiatric disorders as 'circuit disruptions' [3] has stimulated analyses of synaptic regulatory pathways that are dysregulated among neuropsychiatric disorders with shared deficits in executive functions. One molecular pathway notably highlighted is the formation and maturation of dendritic spines whose structural plasticity is tightly coordinated with synaptic function and plasticity [4] and whose morphological abnormalities have been implicated in a number of psychiatric and neurodevelopmental disorders [5, 6], particularly those that involved PFC.

During the postnatal development, PFC is under the pressure of important neuromodulations notably by dopamine (DA). Proper DAergic tone exerts a prominent action in the formation and dynamics of dendritic spines [7] and in controlling synaptic activity and plasticity [8] but also in optimizing executive functions, including working memory and behavioural flexibility [9, 10]. Not surprisingly, aberrant DA levels and inappropriate DAergic neuromodulation of glutamatergic synapses are commonly observed in neuropsychiatric disorders [11, 7]. In the PFC, DA innervation and receptor expression are present early in development, mature during adolescence and form stable patterns in adulthood. This prolonged development timeline provides a large window of 'critical period' during which potential alterations in the mechanisms regulating DA homeostasis can induce a variety of effects including altered spinogenesis and dysfunctional glutamatergic synapses [7, 12] and cognitive dysfunctions [10]. Despite the importance of DA in controlling the development and the functions of the PFC, we are still largely ignoring the cellular mechanisms regulating DA homeostasis and in particular the specific roles that astrocytes may play.

Astrocytes are the most abundant glial cell type of the mammalian brain and are now recognized as central cellular elements controlling synapse formation and maturation [13–16], but also the modulation of many aspects of synapses physiology, network activity, and cognitive functions [17–20]. Whether and how astrocytes may contribute to the homeostasis of DA has never been investigated in detail, although decades of research have established that key enzymes for its metabolism: i.e., mitochondrial enzyme monoamine oxidase B (MAOB) and cathecholamine O methyl transferase (COMT) are mainly expressed in astrocytes [21, 22]. Most interestingly, according to recent transcriptome analyses astrocytes express genes encoding for proteins involved in monoamines transport and storage such as the plasma membrane organic cation transporter 3 (OCT3) [21, 23–25], and, intriguingly, also vesicular monoamine transporter 2 (VMAT2) [21, 25], an integral vesicular membrane protein that in neurosecretory cells directly controls the efficient uptake of cytosolic monoamines into intracellular vesicles [26–28]. Although a novel mRNA splice variant of *Drosophila* VMAT (DVMAT-B) has been found in a small subset of glia in the lamina of the fly's optic lobe [29], to date VMAT2 in mammals is thought to be expressed exclusively in neurons. Here, by studying a possible role for astrocytes in the homeostasis of brain monoamines we find that a subset of cortical astrocytes (i.e., astrocytes located in the frontal and prefrontal cortex- PFC) are endowed with unique features of dopaminergic (DAergic) glial cells insofar as they express VMAT2 together with two key proteins for DA uptake and metabolism (i.e., plasma membrane transporter OCT3 and catabolic enzyme MAOB) and by taking up and metabolizing DA they control DA homeostasis. Importantly, they acquire these DAergic features during the period of postnatal development, when the extracellular levels of DA are crucial for orchestrating spines formation/maturation and thus the dynamic refinement of neuronal circuit connectivity [12, 30]. We find that plasma membrane OCT3 transporter provides effective control of extracellular levels of DA and that VMAT2 directly controls MAOB-dependent metabolism capacity by sequestering DA from the cytoplasm. Indeed, by using in vivo conditional gene inactivation we find that lack of VMAT2-dependent DA storage in astrocytes leads to an aberrant increase in the activity of MAOB and of OCT3 transport and, consequently, to decreased extracellular levels of DA. Unbalanced DA levels in the PFC induce profound alterations of synaptic transmission and plasticity, spine formation and maturation, as well as of cognitive performances, that are reminiscent of developmental psychiatric disorders. We further find that viral-mediated replacement of VMAT2 in astrocytes and/or systemic treatment with L-3,4-dihydroxyphenylalanine (L-DOPA) prevent the onset of cognitive phenotypes, thus providing a causal link between absence of VMAT2 in astrocytes, decreased levels of DA and onset of cognitive deficits. As a whole, we show that, like neurons, astrocytes are integral components of the cellular pathways regulating DA homeostasis in the PFC, a mechanism required for correct synapse patterning during postnatal development and the appropriate maturation of complex cognitive performances involving DA modulation.

## Material and methods

### FACS of astrocytes and semi-quantitative PCR

Glial fibrillary acidic protein/enhanced cyan fluorescent protein (GFAP-ECFP) [31], aldehyde dehydrogenase 1 family, member L1/enhanced green fluorescent protein (ALDH1L1-EGFP), cre^ERT2^XVMAT2XR26-tdTomato and cre^ERT2^XR26-tdTomato transgenic reporter mice were used. Frontal cortex were dissected from P40 old mice and samples were prepared as previously described [21]. CFP, ECFP and EGFP or tdTomato positive astrocytes were purified by fluorescence activated cell sorting (FACS) using a MoFlo AstriosEQ High speed cell sorter. Astrocytes were identified based on high CFP, EGFP and tdTomato fluorescence (see Supplementary Information for details). Total RNA from sorted cells was isolated with RNeasy Mini Kit and the quantitative real-time PCR was done on C1000T Thermal Cycler as already described [32] (see Supplementary Information for details and for primer sequences).

### Tissue preparation, immunohistochemistry and histology

Sprague Dawley rat (P30-40) aVMAT2cKO (P40), control LoxTAM (P40) and R26EYFP^lox/lox^-hGFAPcre^ERT2^ (P40) were deeply anesthetized with sodium pentobarbitone (6mg/100g body wt, i.p.) and immediately perfused intracardiacally with fresh 4% paraformaldehyde in 0.1 M phosphate-buffered saline (pH 7.4). Brains were postfixed overnight, and then equilibrated in 30% sucrose overnight a 4 °C. Sagittal sections (30 μM) were cut at −20 °C using a cryostat and stored at −80 °C (see Supplementary Information for details).

### Lowycril embedding and postembedding immunogold

Immunogold cytochemistry was carried out as described [33] using PFC specimens from adult Wistar rats (P30-P40) fixed by perfusion through the heart (4% formaldehyde and 0.1% glutaraldehyde). Brain sections were cryoprotected in glycerol, frozen in liquid propane, freeze-substituted with methanol, and embedded in Lowicryl HM20. Ultrathin sections of 80 nm were cut from the blocks obtained. The ultrathin sections were processed with the antibodies according to an immunogold procedure previously described elsewhere (see Supplementary Information for details) [34].

### Generation of aVMAT2cKO mice

aVMAT2cKO mice were generated by crossing the hGFAPcre^ERT2^ line expressing a tamoxifen (TAM)-inducible cre recombinase transgene driven by the human astrocytic glial fibrillary acidic protein (hGFAP) promoter [35] with VMAT2^lox/lox^ line containing cre-excisable loxP sequences in the endogenous VMAT2 [36] to create hGFAPcre^ERT2^VMAT2lox/+(F1) mice. The F1 progeny were then crossbred with VMAT2^lox/lox^ mice to create GFAPcre^ERT2^VMAT2^lox/lox^ (F2) and VMAT2^lox/lox^ (F2) mice (Supplementary Figures S2a–c). The F2 progeny (namely CreLox) were injected with 100 mg kg^−1^ TAM or oil as appropriate from P20 to P28 in accordance to Swiss animal guidelines. The controls were VMAT2^lox/lox^ injected with TAM (namely control LoxTAM) and CreLox mice injected with oil/ethanol vehicle (namely control CreLoxOil). The mice used for all of the experiments had a C57BL/6 background. (See Supplementary Information for Maintenance, breeding and genotyping).

### Quantitation of brain monoamines

Monoamines and metabolites were quantified as described previously [37] with some modifications (See Supplementary Information for details).

### Stereotaxic surgery, virus injection and microdialysis

aVMAT2cKO and control LoxTAM mice were anesthetized with isoflurane, and mounted in a stereotaxic apparatus. The viruses used in all of the experiments (lentiVMAT2 and lentiEGFP) were injected bilaterally into the PFC (AP + 2.0 mm, L ± 0.4 mm, DV −2.5 mm) at a rate of 100 nL/min-1 using a Hamilton syringe and CMA400 pump and allowed to incubate for 3–4 weeks before performing the experimental tests (See Supplementary Information for details).

### Time course of dopamine uptake in primary astrocytes

Primary astrocytes isolated from C57BL/6, LoxTAM and aVMAT2cKO pups were prepared as previously described [33, 38] (see Supplementary Information for details) and seeded in a 96-well plate in the incubator at 37 °C and 5% CO2. At the day of the experiment, cells were washed twice with Krebs Ringer Hepes (KRH) buffer and then preincubated for 60 min at 37 °C in KRH buffer in the presence of reserpine (1 μM the presence of deprenyl (1 μM) or vehicle. Afterwards, the cells were incubated for various periods of time with 3 mM of DA using 150 nM of [^3^H]-D dopamine (Dihydroxyphenylethylamine 3,4-[ring-2,5,6–3 H], 60 Ci/mmol) as a tracer (see Supplementary Information for details).

### Quantification of DOPAC and DA in primary astrocytes

(see Supplementary Information for details).

### Electrophysiological slice recording

In all electrophysiological recordings, n indicates the number of independent slices analyzed. Recordings were performed on acute coronal sections of the prefrontal cortex from P45-P60 mice in oxygenated (95% O2/5% CO2) artificial cerebro-spinal fluid. (see Supplementary Information for details).

### Morphological analysis of dendrites and spines

Dendritic spine density and spine morphology was assessed as previously described [39]. For spine and dendrites analysis we used two fluorescent transgenic mice (aVMAT2cKO-Thy1EGFP and control LoxTAM-Thy1EGFP) obtained by the crossbreeding of Thy1EGFP [40] with aVMAT2cKO and VMAT2^lox/lox^, respectively. Confocal microscopy analysis was performed with a Leica confocal imaging system (TCS SP5) with a 40 × (1.8 NA) or with 63x (2.8 NA) oil immersion objectives. (see Supplementary Information for details).

### Cognitive tests

The behavioural phenotyping and cognitive tests were performed using aVMAT2cKO and control LoxTAM mice at P45-P60 (see Supplementary Information for details).

### Statistical analysis

Samples size (*n*) have been indicated in the text (results) and in supplementary materials and methods. They have been based on pilot experiments and previous works. Animals have been characterized in each experiments and randomly allocated to groups. Investigators were blinded to the groups during the experiments (where applicable). All analyses were performed using GraphPad Prism 6.0 software. Then, a one-way ANOVA was performed followed by Bonferroni’s posthoc tests or Tukey posthoc test. For two sample comparisons, unpaired t-test was used. For behaviour and electrophysiology procedures, parametric tests were used: two-way ANOVA with repeated measures followed by a posthoc Tukey’s HSD test. All data showed similar variances and are presented as mean ± SEM. Statistical significant was considered at the *p* < 0.05.

## Results

### Astrocytes in the developing PFC are immunopositive for the determinants of dopamine metabolism

**Fig. 1:**
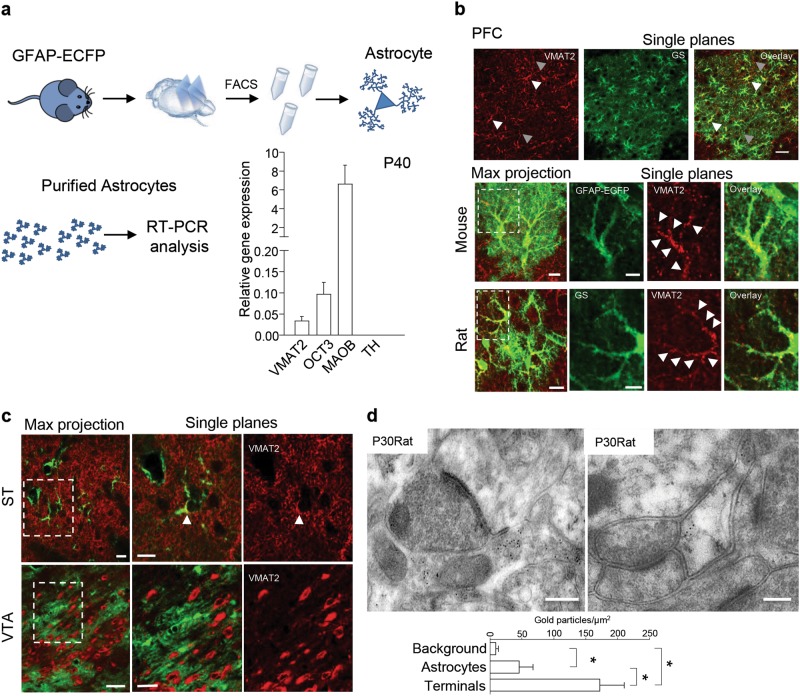
VMAT2 is enriched in astrocytes located in the frontal cortex. **a** Representative image of the RT-PCR analysis and quantification of the relative expression of OCT3, VMAT2, MAOB and TH mRNA in FACS-sorted astrocytes in comparison with β-actin. The error bars indicate the SEM. **b** Representative confocal images show VMAT2 immunolabelling (red) in mouse prefrontal cortex (PFC). Astrocytes are stained with glutamine synthase (GS, green). The VMAT2 signal is highlighted by white arrows in astrocytes and grey arrows in neuronal fibres. Scale bars: 30 μm. High magnifications represent confocal images showing VMAT2 immunolabelling (red) in the PFC of mouse and rat (P30-40). Scale bars: 5 μm. **c** Confocal sections showing VMAT2 (red) in the ST and VTA of rats; the astrocytes are stained with GS (green). Note the absence of VMAT2 immunolabeling in the GS-positive astrocytes of the VTA . In the ST, the VMAT2 signal in astrocytes is highlighted by white arrows. Scale bars: 20 μm. **d** Electron microscopy sections show the immunogold labelling of VMAT2 in L5 of a P30 rat. On the left: immunogold particles for VMAT2 in a dopaminergic axonal bouton making synapse with an asymmetric synapse (synaptic triad). VMAT2, 10 nm gold particles . On the right: immunogold particles for VMAT2 located in a peri-synaptic astrocytic process. VMAT2, 10 nm gold particles . Scale bars: 250 nm. Histograms show the average density of VMAT2 immunogold particles in astrocytic processes and axonal boutons in L5 of rat PFC. Note that, although lower than that of dopaminergic boutons (−73%, *n* = 3), the density of the VMAT2 immunogold particles is significantly higher (+82%, *n* = 3) than the background calculated on mitochondria. The error bars indicate the SEM

The presence of bona fide genes involved in the synthesis, uptake, storage and degradation of DA was here investigated in purified astrocytes micro-dissected, postnatal day 40 (P40) frontal cortex by means of FACS and GFAP-ECFP [31] or ALDH1L1-EGFP [32] transgene reporter mice. We detected significant levels of RNA for the plasma membrane transporter OCT3, the metabolic enzyme MAOB and the vesicular transporter VMAT2 (Fig. 1a, Supplementary FigS1a), but could not find any signal for tyrosine hydroxylase (TH), an enzyme that is essential for the production of monoamines. Analysis of the purified ECFP- and of EGFP-positive astrocytes (sorting to yield > 99% pure positive cells) revealed background levels of the markers of neurons, dendrites or oligodendrocytes (*n* = 11 mice, Supplementary Figure S1b, c), thus validating the purity of the cell extracts. Further validation was provided by the fact that immunolabelling experiments performed with previously validated polyclonal antibody raised against OCT3, MAOB, VMAT2, and TH (Supplementary Table 1) detected the presence of all of these proteins in vivo in peri-adolescent rodents [P30-40] (Supplementary Figures S1d–f Fig. 1b). The VMAT2 signal was readily recognizable in the cell bodies and large processes of glutamine synthase (GS)-positive astrocytes located in the frontal regions (including the prefrontal cortex—PFC) (Fig. 1b), but the signal was respectively weak or virtually absent in the striatum (ST) and ventral tegmental area (VTA) (Fig. 1c). These observations were confirmed by the similar results obtained in P30 wt and BAC transgenic hemagglutinin(HA)-tagged VMAT2 mice (Supplementary Figure S1g–h), in which the expression of HA epitope-tagged VMAT2 revealed HA instead of VMAT2. Immunostaining for VMAT2 and GS of specimens taken from mice at different times during the first four postnatal weeks (P7, P14, P30) showed moreover that VMAT2 signal in GS-positive astrocytes arose in the second postnatal week (Supplementary Figure S1i). Several studies have suggested that transgenic reporter lines for ALDH1L1-EGFP can be used to identify astrocytes in mature CNS . In fact, immunostaining analysis confirmed that ALDH1L1-EGFP positive cells in PFC co-labelled extensively (95–98%) with GS positive mature astrocytes (Supplementary Figure S1j). Western blot (WB) analysis of brain tissues obtained from wild-type (wt), VMAT2 knock-out (VMAT2^−/−^) and heterozygote VMAT2 (VMAT2^+/−^) mice [41] and immunostaining of VTA in wt and VMAT2^−/−^ brain tissues at P5 confirmed that the detected VMAT2 signal was specific as no signal was observed in the VMAT2^−/−^ mice (*n* = 3 mice each group for WB and *n* = 3 mice for immunostaining, Supplementary Figures S1k–n). Consistent with these findings, quantitative immunogold analyses confirmed that a well characterized VMAT2 signal (Supplementary Figure S1o-q), was present in the layer 5 (L5) mPFC astrocytic processes of P30 rats recognized for the expression of GLT1/GLAST25 or GS (Fig. 1d; Supplementary Figure S1r), although the density of gold particles was about 3 fold less than in DAergic axons but still significantly above background (+82%) calculated in mitochondria (*n* = 3 rats; one-way ANOVA, Bonferroni’s post-test correction, *p < 0.05). Taken together, these findings indicate that OCT3, VMAT2 and MAOB are present in PFC astrocytes and may contribute to the regulation of DA homeostasis.

### Conditional knock-out mice with the selective deletion of VMAT2 in astrocytes

In order to investigate the role of VMAT2 in DA homeostasis, we generated an inducible knock-out mouse line in which the protein could be specifically deleted in a temporally controlled manner by crossing mice harbouring the tamoxifen(TAM)-inducible *cre*^*ERT2*^ recombinase transgene driven by the hGFAP promoter (hGFAPCre mice) [35, 42] with mice containing *cre*-excisable *loxP* sequences in the endogenous *VMAT2* gene (VMAT2^lox/lox^ mice) [36].

**Fig. 2:**
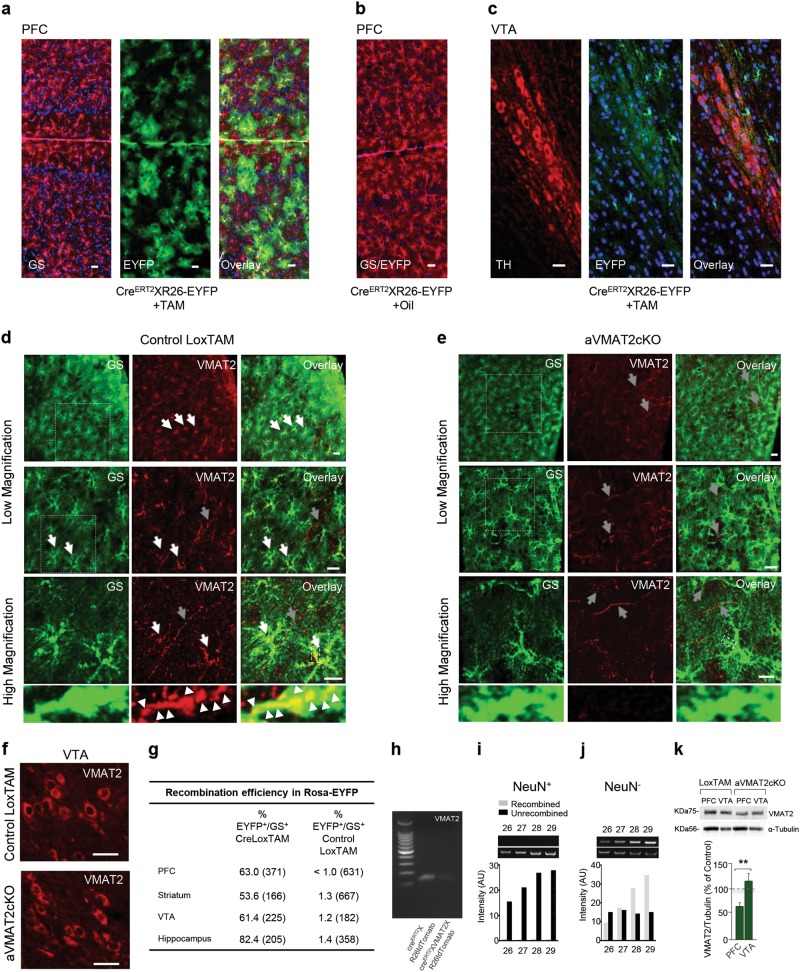
Selective and inducible deletion of astrocyte VMAT2 in aVMAT2cKO mice. **a**–**c** Confocal sections show TAM-induced recombination in the prefrontal cortex (PFC) and ventral tegmental area (VTA) of Cre^ERT2^XROSA(R)26-EYFP mice reporter on (P40). **a** EYFP immunolabelling (green, enhanced by anti-GFP immunostaining) is confined in the glutamine synthase (GS)-positive astrocytes (red) in the PFC (P40); the nuclei are stained with DAPI (blue). The efficacy of TAM-induced recombination in astrocytes is 63%, as evaluated by the expression of EYFP in GS-positive cells. Scale bar: 50 μm. **b** EYFP immunolabelling (green) is not detectable in the PFC of the Cre^ERT2^XR26-EYFP mouse treated with ethanol/oil (P40). The astrocytes are stained with GS (red). Scale bar: 50 μm. **c** EYFP immunolabelling (green) is confined to the VTA astrocytes of recombined Cre^ERT2^XR26-EYFP mice (P40). Tyrosine hydroxylase (TH, red) is used as a marker of DAergic neurons. Note that no recombination occurs in the DAergic neurons, as shown by the absence of EYFP immunolabelling in the TH-positive cell bodies. Nuclei are stained with DAPI (blue). Scale bar: 50 μm. **d** and **e** Top: Confocal sections (low magnification) of VMAT2 immunolabelling (red) in the PFC of P40 control LoxTAM and recombined aVMAT2cKO mice. Bottom: magnified images of VMAT2 immunolabelling (red) in the PFC of recombined aVMAT2cKO and control LoxTAM mice. Note that VMAT2 immunolabelling is no longer detectable in the GS-positive cells of the recombined aVMAT2cKO mice. Scale bars: 20 μm. **f** VMAT2 immunolabelling in the VTA of control LoxTAM mice and recombined aVMAT2cKO (P40). Note that VMAT2 immunolabelling in VTA neurons is not affected by the TAM-induced recombination. Scale bars: 20 μm. **g** Table of recombination efficiency in Rosa-EYFP mice. The recombination is calculated in control LoxTAM-ROSA26-EYFP and Cre^ERT2^XROSA26-EYFP mice (P40). **h** Representative image of semi-quantitative PCR analysis of mRNA levels for VMAT2 measured in FACS sorted astrocytes from control Cre^ERT2^XR26-tdtomato and double floxed Cre^ERT2^XVMAT2XR26-tdtomato mice. **i** and **j** Semi-quantitative PCR analysis of DNA isolated from FACS-sorted NeuN positive (+) and negative (-) cells from recombined aVMAT2cKO brains (P40). The histograms show the average number of arbitrary units (AUs) of DNA amplified from NeuN+ neurons and other NeuN- brain cells in recombined (grey) and unrecombined DNA (black). **k** Western blot analysis of VMAT2 expression in the PFC and VTA of P40 aVMAT2cKO and control LoxTAM mice (P40). The error bars indicate the SEM

The progeny inheriting both alleles (CreLox, Supplementary Figures S2a–c) and treated with TAM from P20 to P28 presented the selective deletion of astrocyte VMAT2, and are here referred to as aVMAT2cKO mice. The controls were VMAT2^lox/lox^ mice injected with TAM (control LoxTAM), and CreLox mice injected with the oil/ethanol vehicle (control CreLoxOil). The aVMAT2cKO mice were viable, their body weight was normal, and their overall brain morphology did not reveal any gross anatomical difference or any sign of apoptosis or oxidative DNA damage (Supplementary Figures S2d–f); staining with Nissl and NeuN revealed a normal frequency of cells and normal layering in the frontal cortex (Supplementary Figures S2g,h), and the fact that WB analysis revealed no difference in GFAP excluded any reactive gliosis (Supplementary Figure S2i). The specificity and efficacy of TAM-induced astrocyte VMAT2 excision was confirmed in the fluorescent Cre^ERT2^XR26-EYFP-reporter [43] and aVMAT2cKO mice lines. Confocal microscopy revealed the expression of EYFP in astrocytes in different brain areas, and showed recombination in 63% of PFC astrocytes (*n* = 4 mice; Figs. 2a, g); no recombination was detected in the astrocytes of the oil-treated Cre^ERT2^XR26-EYFP mice (Fig. 2b) or in neurons of the PFC (Supplementary Figure S2j, k) and DAergic neurons of the VTA (*n* = 4 mice; Fig. 2c). Additionally, mRNA levels for VMAT2 were significantly decreased in FACS-sorted astrocytes from fluorescent double floxed Cre^ERT2^XVMAT2XR26-tdTomato mice with respect to those purified from control Cre^ERT2^XR26-tdTomato mice (−83.5 ± 2.5%, *n* = 3 mice, Student's *t-*test, **p < 0.001; Fig. 2h) and recombination was absent in neurons of whole brain, as confirmed by immunolabelling or polymerase chain reaction (PCR) analysis of the recombined stop sequence (*n* = 27 mice; Figs. 2i, j; Supplementary Figures S2l-s). Immunostaining for VMAT2 confirmed the loss of the VMAT2 signal in PFC astrocytes but not the DAergic projections (Figs. 2d, e) or VTA DAergic neurons (Fig. 2f). Accordingly, there was a significant reduction in VMAT2 protein expression in the PFC (−30%) but not in the VTA of the aVMAT2cKO mice (*n* = 3 mice, Student's *t* test, ***p < 0.001; Fig. 2k), thus supporting our observation that VTA astrocytes do not express VMAT2 and ruling out any leakage of recombination to neurons. Thus, aVMAT2cKO mice are a valid model for investigating the role of astrocyte in the regulation of monoamine levels in the postnatal brain.

### Deletion of astrocyte VMAT2 disrupts DA metabolism in the PFC

**Fig. 3:**
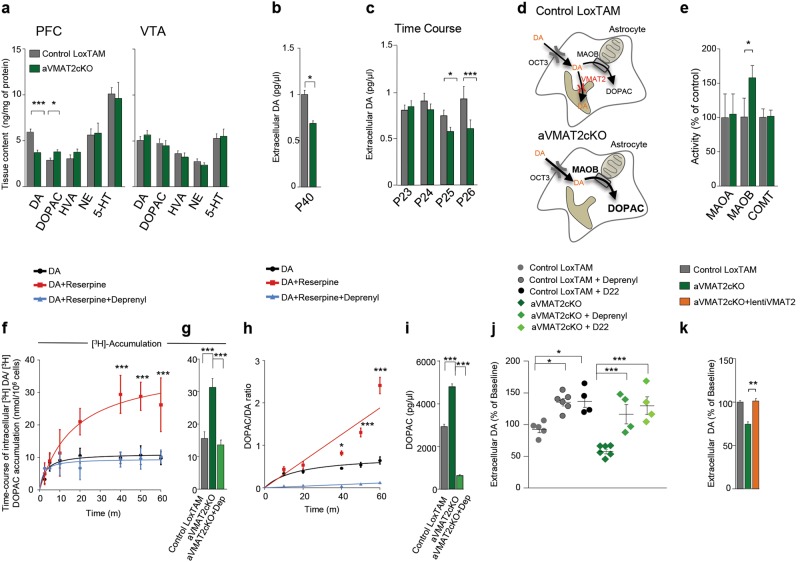
Astrocyte VMAT2 deletion disrupts DA metabolism and leads a decrease in dopamine levels in the PFC. **a** HPLC quantification of total dopamine (DA), 3,4-dihydroxyphenylacetic acid (DOPAC), homovanillic acid (HVA), norepinephrine (NE) and serotonin (5-HT) (ng per mg of protein) in the PFC and VTA of recombined aVMAT2cKO and control LoxTAM mice (P40). The error bars indicate the SEM. **b** Histograms show the average basal extracellular levels of DA calculated in the extracellular perfusates of the in vivo microdialysis in control LoxTAM and recombined aVMAT2cKO mice (P40). The microdialysis probes were placed in the PFC, and DA levels (pg/μl) were measured at baseline for 30 min. The error bars indicate the SEM. **c** Histograms show the average basal extracellular levels of DA calculated in the extracellular perfusates of the in vivo microdialysis of control LoxTAM (grey) and recombined aVMAT2cKO mice (green) at different time points after the first TAM injection. The microdialysis probes were placed in the PFC, and DA levels (pg/μl) were measured at baseline for 150 min. The error bars indicate the SEM. **d** Diagram shows the contribution of astrocytic VMAT2 to DA metabolism. **e** Histograms shows enzymatic activities of MAOA, MAOB and COMT measured in PFC brain homogenates of control LoxTAM and recombined aVMAT2cKO mice (P40). The error bars indicate the SEM. **f** Dopamine accumulation in wt-derived primary cultured astrocytes using [^3^H]-Dopamine. Curves represent time–dependent specific accumulation of [^3^H]-Dopamine (black curve, 3 mM DA and 150 nM of [^3^H]-Dopamine as a tracer) in cultured astrocytes incubated with reserpine (red curve) or reserpine plus deprenyl (blue curve). Note that in the presence of reserpine the accumulation of [^3^H] is the sum of [^3^H]-Dopamine and [^3^H]-DOPAC. The error bars indicate the SEM. **g** Histograms shows [^3^H]-Dopamine accumulation in primary cultured astrocytes derived from control LoxTAM and recombinant aVMAT2cKO mice in the presence or in the absence of deprenyl (1 μM). The error bars indicate the SEM. **h** Time-course effect of reserpine (1 μM) and deprenyl (1 μM) on the intracellular levels of DOPAC/DA ratio calculated in LoxTAM or aVMAT2cKO-derived primary cultured astrocyte. Cultured astrocytes were incubated with DA (3 mM) in presence or absence (black curve) of reserpine (red curve) and reserpine plus deprenyl (blue curve) for 10, 20, 40, 50 and 60 min. Note that in the presence of reserpine there is an increased accumulation of DOPAC. The error bars indicate the SEM. **i** Histograms show intracellular levels of DOPAC in cultured astrocytes derived from control LoxTAM and recombinant aVMAT2cKO mice. Cultured astrocytes were incubated with DA (3 mM) in presence or absence of deprenyl (1 μM) for 40 min. The error bars indicate the SEM. **j** Graph shows the average levels of DA calculated in extracellular perfusates of the in vivo microdialysis in control LoxTAM and recombined aVMAT2cKO mice (P40) treated with deprenyl (10 mg/kg, i.p.) or D22 (1,1-diethyl-2,2-cyanine iodide, 100 μM). The microdialysis probes were placed in the PFC, and DA levels (pg/μL) were measured at baseline for 30 min. The error bars indicate the SEM. **k** Histograms shows the average levels of DA calculated in extracellular perfusates of the in vivo microdialysis in the PFC of control LoxTAM and recombined aVMAT2cKO mice locally infected with astrocyte-targeted with lentiGFP or lentiVMAT2viruses, respectively (P40). Data expressed as fold percentages of baseline levels. The error bars indicate the SEM

We next assessed potential changes of monoamine transmission in aVMAT2cKO mice. In tissue homogenates of the PFC obtained from aVMAT2cKO mice we found a significant decrease in total DA content of ~38% in the PFC but not the VTA (*n* = 6 mice each group, Student’s *t-* test, ***p < 0.001; *p < 0.05; Fig. 3a). Interestingly, this effect was DA specific as there was no change in serotonin (5-HT) or norepinephrine (NE) content; there was also no significant alteration in the levels of DA and the other catecholamines in the VTA, thus confirming that the astrocytes in the VTA did not contain VMAT2. We also detected a selective ~25% increase in its 3,4-dihydroxyphenylacetic acid (DOPAC) metabolite, in line with the previously reported increased cytosolic turnover of DA in VMAT2 full knock out mice [44]. Further in vivo microdialysis analyses confirmed that deleting astroglial VMAT2 led to a ~ 23 and ~ 33% reduction in extracellular DA levels in the PFC of P25 and P40 mice, respectively (*n* = 6 mice, Student’s *t-* test, *p < 0.05; Fig. 3b; *n* = 3 mice each group, per day, Student’s *t-* test, ***p < 0.001; *p < 0.05; Fig. 3c). The ~30% decrease in tissue content of DA induced by deleting VMAT2 from astrocytes and the concomitant increase in the MAOB metabolite DOPAC suggested rapid metabolism of the excess cytosolic DA that could not be stored in the absence of VMAT2 (Fig. 3d). In line with these hypothesis, we found a selective increase in MAOB activity in the PFC of aVMAT2cKO mice in comparison with control mice (about +57%; *n* = 5 mice each group, Student’s t- test, *p < 0.05; Fig. 3e). We then investigated the importance of the plasma membrane OCT3 transporter and MAOB activity in regulating extracellular DA clearance by performing two different series of experiments.

First, we examined the time course of DA accumulation and of DA turnover (i.e., DOPAC/DA ratio) in primary cultured astrocytes (wt) in the absence/presence of the specific VMAT2 blocker reserpine (1 µM), and in primary cultured astrocytes derived from control LoxTAM or aVMAT2cKO mice (Figs. 3f–i). Curves in Supplementary Figure S3a show that astrocytes accumulated ^3^H (DA 3 mM, ^3^H-DA 150 nM) in a time-dependent manner and that the uptake was inhibited at all time points by the specific OCT3 inhibitor D22 (5 µM). In the absence of VMAT2 inhibitor reserpine, both ^3^H accumulation and DOPAC/DA ratio reach the plateau in about 15 min (Figs. 3f, h), suggesting that DA taken up by astrocytes accumulates into the cytoplasm (as well as into organelles expressing VMAT2) thus stopping the uptake and, consequently the degradation of DA into DOPAC. However, inhibition of VMAT2 with reserpine significantly increases the cytosolic DA turnover measured as DOPAC/DA ratio (Fig. 3h red curve, +77.7 ± 12%, +141.63 ± 19.23% and +276.56 ± 29.87% at 40, 50 and 60 min, respectively, two-way ANOVA *F* = 29.27, *p* < 0.001 followed by Bonferroni’s post-hoc correction, ***p < 0.001; *p < 0.05) and DOPAC levels increase over time without reaching a plateau, suggesting that DA taken up by astrocytes is continuously degraded, thus driving the uptake activity. Consequently, the total amount of cytosolic ^3^H-accumulation (i.e., ^3^H-DA taken up and ^3^H-DOPAC resulted by degradation of ^3^H-DA, Fig. 3f) increases over the time, does not reach a real plateau and is significantly different from control at 40, 50 and 60 min (red curve; 29.40 ± 5.83 vs 9.86 ± 1.723 at 40 min, 28.89 ± 4.322 vs 10.71 ± 2.845 at 50 min, 31.44 ± 7.842 vs 9.78 ± 1.9776 at 60 min, two-way ANOVA *F* = 14.21, *p* < 0.001 followed by Bonferroni’s post-hoc correction, ***p < 0.001). The role of MAOB in the increase of intracellular DOPAC levels and ^3^H accumulation (i.e., of total amount of DA taken up by astrocytes), was highlighted by administrating reserpine in the presence of deprenyl (Figs. 3f, h, blue curves; 9.35 ± 2.317 vs 29.40 ± 5.83 at 40 min, 9.63 ± 3.233 vs 28.89 ± 4.322 at 50 min, 10.30 ± 1.995 vs 31.44 ± 7.842 at 60 min, two-way ANOVA *F* = 14.21, *p* < 0.001 followed by Bonferroni’s post-hoc correction, ***p < 0.001; *p < 0.05). Similar results have been obtained by evaluating ^3^H-accumulation and DA turnover in cultured cells derived from aVMAT2cKO mice, where VMAT2 was genetically deleted (Figs. 3g; 15.57 ± 2.071 LoxTAM, 31.29 ± 2.7 aVMAT2cKO, 13.66 ± 1.44 aVMAT2cKO + deprenyl, one way ANOVA *F* = 18.80, ***p < 0.001 followed by Tukey’s post-hoc test; Fig. 3i  163.40 ± 4.67% aVMAT2cKO, one way ANOVA *F* = 388.1, ***p < 0.001 followed by Tukey’s post-hoc test). Overall, these results suggested that in the absence of VMAT2 the incessant degradation of DA by MAOB, by keeping low cytosolic concentrations of DA, droves the activity of the plasma membrane OCT3 transporter and, consequently decreases the extracellular levels of DA (Fig. 3d).

**Fig. 4:**
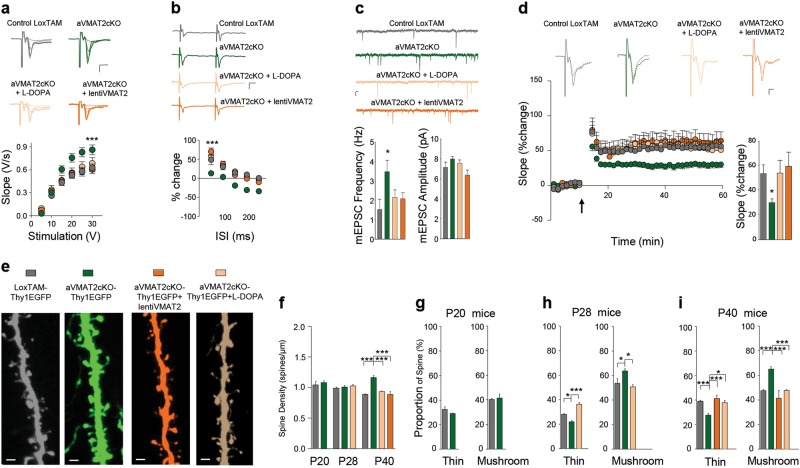
Astrocyte VMAT2 deletion leads to synapse impairments. **a** Top: Example traces shows superimposed fEPSP as stimulation intensity increases. Calibration bars: 5 ms, 0.5 mV. Bottom: Graph  shows the maximum EPSP slope plotted against stimulus. The PFC slices prepared from aVMAT2cKO mice (green) show enhanced basal synaptic transmission with respect of control LoxTAM mice (grey) (P45-60). The enhanced basal synaptic transmission  in aVMAT2cKO mice is rescued with astrocyte-targeted lentiviral vectors encoding VMAT2 (lentiVMAT2) (orange) or with chronic treatment with L-DOPA/benserazide (20 mg/kg + 12.5 mg/kg) (pink). **b** Top: Example traces showing facilitation at 50 ms ISI. Calibration bars: 10 ms, 0.5mV. Bottom: Graph shows pooled paired-pulse ratios (slope2/slope1 x100 −100) plotted against the inter-stimuli interval (ISI). The deletion of astrocyte VMAT2 leads to a clear deficit in short-term facilitation, which indicates an increased probability of release. Note that the deficit is rescued by lentiVMAT2 (orange) and L-DOPA (pink). **c** Top: Representative traces of the intracellular recording of L5  pyramidal neurons. Bottom: Histograms showing that mEPSC frequency is increased in mice lacking astrocyte VMAT2 (lower left panel, green), whereas mEPSC amplitude remains unchanged. Note that the increased mEPSC frequency is rescued by lentiVMAT2 (orange) and L-DOPA/benserazide (pink) (lower right panel). **d** The administration of tetanic stimulation (6 x 50 Hz, 2 s) is indicated by an arrow and the baseline by a dashed line. Representative traces showing responses before (dashed line) and 60 min after tetanus delivery (bold line). Calibration bars: 5 ms, 0.2 mV. LTP is significantly reduced in aVMAT2cKO mice 60 min after induction. Note that LTP is rescued by lentiVMAT2 (orange) and L-DOPA/benserazide (pink). **e** Representative confocal images showing L5 PFC dendritic spines in recombined aVMAT2cKO-ThyEGFP (green), recombined aVMAT2cKO-ThyEGFP + lentiVMAT2 (orange), recombined aVMAT2cKO-ThyEGFP + L-DOPA/benserazide (pink), and control LoxTAM-ThyEGFP mice (grey) (P40). Scale bar: 1 μm **f** Histograms of spine density calculated in L5 of the PFC of recombined aVMAT2cKO-ThyEGFP (green), recombined aVMAT2cKO-ThyEGFP + lentiVMAT2 (orange), recombined aVMAT2cKO-ThyEGFP + L-DOPA/benserazide (pink), and control LoxTAM-ThyEGFP mice (grey) at three developmental stages (P20, P28, P40) (*n* = 3 mice per group). The error bars indicate the SEM. **g**–**i** Histograms showing the proportion of thin and mushroom spines in L5 of the PFC of recombined aVMAT2cKO-ThyEGFP (green), recombined aVMAT2cKO-ThyEGFP + lentiVMAT2 (orange), recombined aVMAT2cKO-ThyEGFP + L-DOPA/benserazide (pink), and control LoxTAM-ThyEGFP mice (grey) at three developmental stages (P20, P28, P40). The error bars indicate the SEM

Second, we applied deprenyl (10 mg/kg, i.p.) and D22 (local application, 100 μM) [45] in control and aVMAT2cKO mice, and measured extracellular DA levels by means of in vivo microdialysis (Fig. 3j; Supplementary Figure S3b). We found that both deprenyl and D22 significantly increased the levels of DA by about 40% in control LoxTAM mice, and completely restored to wild-type levels when injected into aVMAT2cKO mice (*n* = 6 control mice; *n* = 4 aVMAT2cKO mice, one-way ANOVA *F* = 14.74, *p* = 0.0001 followed by Tukey’s HSD posthoc test, ***p < 0.001; *p < 0.05, Fig. 3j) thus providing strong evidence that DA uptake and degradation by astrocytes caused the reduction in extracellular DA levels observed in the absence of VMAT2.

Finally, in order to confirm the prominent role of astroglial VMAT2 in modulating DA levels, we generated a lentiviral vector that enabled the selective re-expression of VMAT2 in astrocytes [46] and validated this expression using a lentivirus bearing green fluorescent protein (GFP) (*n* = 5 mice; Supplementary Figures S3c–h). The stereotactic injection of the VMAT2 lentivirus in the PFC of P25 aVMAT2cKO mice induced the re-expression of VMAT2, as demonstrated by means of WB analysis (Supplementary Figure S3c) and, remarkably, fully rescued basal extracellular DA levels in the PFC (*n* = 4 mice each group, one way ANOVA *F* = 34, ***p < 0.0001 followed by Tukey’s posthoc test; Fig. 3k). We concluded that astrocytic VMAT2 is necessary to maintain proper DA levels in the developing PFC.

Taken together, the above results show that VMAT2 acts in concert with OCT3 to control cytoplasmic storage and metabolic capacity, and provide the first evidence that astrocytes control DA homeostasis in the PFC.

### Deletion of astroglial VMAT2 does not impair the activity of DAergic neurons

We excluded the possibility of defects in neuronal DA production or release in aVMAT2cKO mice with a series of control experiments. We started by investigating TH, the rate-limiting enzyme in neuronal DA biosynthesis. We did not find any difference in the number of TH-positive projections in L2/3 and L5 of the mPFC of aVMAT2cKO and control mice (Supplementary Figure S4a), and this was confirmed by WB analysis (*n* = 4 mice, Student’s *t* test; Supplementary Figure S4b). We also tested whether the deletion of astroglial VMAT2 could have altered VTA neuron activity by performing single-unit recordings to monitor the in vivo firing rate of VTA DAergic neurons, and did not find any significant differences in the frequency of tonic and phasic spiking (*n* = 7 mice each group, *n* = 23 LoxTAM and 17 aVMATcKO cells analysed. Firing rate 5.03 ± 0.57 vs 4.54 ± 0.47 Hz; unpaired student t-test, t38 = 0.631, *p* = 0.531; Spikes in burst 35.36 ± 6.35 vs 28.78 ± 7.11%, unpaired student *t*-test, t38 = 0.69, *p* = 0.49; coefficient of variation 63.3 ± 5.85 vs 75.22 ± 9.64%, unpaired student *t*-test, t38 = 1.113, *p* = 0.27; Supplementary Figure S4c). Finally, we tested possible defects in neuronal DA release in the PFC by optogenetically stimulating VTA DA neurons while measuring the levels of DA in the extracellular perfusates of the PFC by means of in vivo microdialysis. To this end, a viral (AAV) vector carrying the gene encoding channelrhodopsin-2 (ChR2) and yellow fluorescent protein (EYFP) [47] was stereotaxically introduced in the VTA of control and aVMAT2cKO mice at P40 (Supplementary Figure S4d). ChR2-EYFP expression was evident 15 days after the injection and was restricted to the DA neurons, as shown by TH immunostaning (Supplementary Figure S4e). In vivo, we stimulated VTA DA neurons with high-frequency light trains to evoke phasic DA neuron firing [48, 49] and found that the increased extracellular levels of DA in the PFC were unaltered in aVMAT2cKO with respect to control mice (*n* = 6 mice each group, Student’s *t* test; *p* = 0.9159; Supplementary Figure S4f).

### Decreased DA levels by VMAT2 deletion in astrocytes alters excitatory synaptic transmission and plasticity in the PFC

During postnatal development, DA acting through DA receptors regulates excitatory glutamatergic synapses [8, 30]. We therefore assessed the effects of insufficient extracellular DA levels on excitatory synaptic transmission in the mPFC of aVMAT2cKO mice by first analysing the properties of the top-down PFC pathways thought to contribute to the frontal cognitive processes that are crucially modulated by DA [50].

We used field recordings to characterize basal intracortical neurotransmission in L5 in response to stimulation of the border of L2 [51]. Thus, typical field excitatory post-synaptic potentials (fEPSPs) were evoked in L5 (Fig. 4a; Supplementary Figure S5a) whose initial linear down-slope can be used as an index of synaptic efficacy. Basal synaptic transmission was assessed by recording the input/output (I/O) curves vs. gradually increased stimulation intensities. Analysis of the I/O revealed that synaptic efficacy was significantly increased in aVMAT2cKO mice (*n* = 25) over-control LoxTAM mice (*n* = 23, two-way ANOVA, ***p < 0.001; Fig. 4a) with no changes in cell excitability (Supplementary Figure S5a; Supplementary Figures S5e, f).

To test the pre- vs. post-synaptic locus of this aberrant electrophysiological signature in the aVMAT2cKO mice, we next used paired-pulse protocol to assess pre-synaptic function and short-term plasticity. Paired-pulse faciliatation (PPF) was reduced in aVMAT2cKO mice (*n* = 25) in comparison with control LOXTAM mice (*n* = 23, two-way ANOVA, ***p < 0.001; Fig. 4b; Supplementary Figure S5b), thus suggesting that the probability of neurotransmitter release is increased upon deletion of VMAT2 in astrocytes.

Such scenario was confirmed by whole cell patch clamp recordings of miniature excitatory post-synaptic currents (mEPSCs). Indeed, excitatory synapses onto L5 pyramidal neurons showed a selective increased in the frequency but not the amplitude of mEPSCs in the aVMAT2cKO mice (*n* = 7 slices from 4 control LoxTAM mice: 1.54 ± 0.51, *n* = 13 slices from 2 aVMAT2cKO mice: 3.47 ± 0.57, two-way ANOVA, **p < 0.01; Fig. 4c; Supplementary Figure S5d).

Because PFC neurons typically receive trains of inputs from neighbouring cells in the gamma range of 30–80 Hz during the delay period of working memory tasks, we determined the effects of astrocytic VMAT2 deletion on long-term synaptic plasticity in response to trains of inputs within this physiological frequency range. In control LoxTAM mice, 50 Hz tetanic stimulation enhanced the excitatory synaptic response (fEPSP), resulting in long-term potentiation (LTP) that reached ~60% of its initial level 50–60 min after tetanisation. In contrast, the magnitude of 50 Hz LTP in aVMAT2cKO mice was significantly reduced (+29.21 ± 4.46%, *n* = 21 vs +54.48 ± 6.55%, two-way ANOVA, **p < 0.01; Fig. 4d; Supplementary Figure S5c).

As astrocyte-specific lentiVMAT2 injected in the mPFC on P25 could restore the extracellular DA levels in the mPFC of aVMAT2cKO mice, we investigated whether this approach could also reverse the synaptic deficits we observed in aVMAT2cKO mice. As shown in Fig. 4, the injection of the lentiVMAT2 was able to rescue basal synaptic transmission (fEPSPs, frequency of mEPSCs, *n* = 13 slices from 3 aVMAT2cKO+lentiVMAT2 mice, two-way ANOVA, **p < 0.01) as well as short- and long-term plasticity in aVMAT2cKO mice to control levels (*n* = 12 slices from 3 and *n* = 10 slices from 4 aVMAT2cKO+lentiVMAT2 mice, two-way ANOVA, *p < 0.01). The results thus confirm the specific involvement of astrocyte VMAT2 in the regulation of excitatory synaptic transmission and plasticity in developing mPFC.

In order to address more directly the role of decreased DA levels in the synaptic signature observed in aVMAT2cKO mice, we performed two types of experiments. We first chronically treated aVMAT2cKO mice with levodopa (L-DOPA) 20 mg kg^−1^ i.p. from P20 to P40, the main clinical relevant treatment that increases DA concentrations. As shown in Fig. 4, L-DOPA treatment not only restored DA levels (Supplementary Figure S3b) but also rescued all synaptic features in L5 of the mPFC (*n* = 9–17 from 4 aVMAT2cKO + L-DOPA mice for basal synaptic activity, short- and long-term plasticity, respectively, two-way ANOVA, ***p < 0.001). Overall, these findings show that astrocytic VMAT2 plays an important role in the modulation by DA of excitatory activity in the developing mPFC.

These changes are chronic and do not imply that acute effects of DA are maintained in the chronically treated animals. Therefore, we next ask whether the normal DA pharmacological modulation of fEPSPs is maintained despite chronic manipulations. Bath application of high concentrations of DA (100 µM) reduced fEPSPs in aVMAT2cKO mice by 36.01 ± 7.07% (*n* = 8, Supplementary Figure S5g) thus reaching control levels (I/O aVMAT2 + DA vs I/O LoxTAM *p* = 0.21, two-way ANOVA), and simultaneously restores PPF (aVMAT2cKO: −9.86 ± 12.13%, aVMAT2cKO + DA: 73.26 ± 32.86% vs. control: 67.36 ± 25.94%) as well as LTP (aVMAT2cKO + DA: +47.88 ± 11.52% vs LoxTAM control mice, *n* = 6, Fig. 4 and Supplementary Figure S5g). These observations confirmed that the normal modulation by DA of neuronal network activity is maintained in the aVMAT2cKO mice and suggest that the physiological features in these mice are caused by the loss of DA inhibition. Positive DA neuromodulation is classically attributed to D_1_-type receptors activation whereas negative modulation relies on D_2_-type receptors activation [8, 52]. As expected, application of the broad-spectrum D_1_/D_2_ receptors antagonist fluphenazine (10–30 µM) did not affect either synaptic transmission, PPF or LTP (data not shown). We then tested the hypothesis that the impaired physiological features in aVMAT2cKO mice were, in fact, mediated by D_2_ receptors. Sulpiride (50 µM), a more selective D_2_R antagonist [53, 54] induces a moderate but significant increase of fEPSPs in wild-type (+32.88 ± 11.71%, *n* = 8), LoxTAM (+27.32 ± 12.04%, *n* = 6), aVMAT2cKO + L-DOPA (+34.38 ± 15.22%, *n* = 10) or aVMAT2cKO + lentiVMAT2 mice (+30.54 ± 6.30%, *n* = 6) (Supplementary Figure S5h-l) after 40 min application. As a consequence, blockade of D2R with sulpiride significantly reduced PPF and LTP in all groups (Supplementary Figure S5h–l). These results revealed that normally astrocytic-derived DA tonically suppresses excitatory synaptic transmission by acting at D2R and that the impairments of excitatory synapses activity upon deletion of VMAT2 in astrocytes resulted from disinhibition of DA input onto pyramidal cells.

### Decreased DA levels by VMAT2 deletion in astrocytes increases spine maturation in the developing PFC

During the maturation of brain circuits that occurs after birth, DA neuromodulation is essential for the development of correct excitatory synaptic connections [30] . We thus investigated whether the increased excitatory synaptic transmission in L5 of the PFC of adolescent aVMAT2cKO mice led to alterations in spine formation and maturation. This was accomplished using a loss-of-function strategy to evaluate the effects of astrocyte VMAT2 deletion by cross-breeding Thy1EGFP fluorescent mice [40] with aVMAT2cKO and control LoxTAM mice. The development of dendritic spine structure in L5 pyramidal neurons was visualized in the resulting fluorescent aVMAT2cKO-Thy1EGFP and control LoxTAM-Thy1EGFP mice by means of anti-GFP immunostaining during the period from the start of TAM treatment on P20 to P40 (Fig. 4e). Before (P20) and 8 days after TAM treatment (P28), the VMAT2-deficient ThyEGFP mice showed normal dendritic spine density in comparison with control mice, whereas the dendritic spine density of L5 pyramidal neurons had substantially increased by P40 (*n* = 3 mice each group, one-way ANOVA *F* = 14.67, *p* = 0.0004 followed by Tukey’s HSD posthoc test, ***p < 0.001; Fig. 4f). Interestingly, starting from P28, the absence of astrocyte VMAT2 significantly increased the number of dendritic spines with larger-diameter heads and short necks (*n* = 4 each group, one-way ANOVA followed by Tukey’s HSD posthoc test, *p < 0.05; ***p < 0.001; Figs. 4g-i, Supplementary Figures S5m–o), a characteristic of mushroom spines [55] and mature synapses[56]. This alteration led to more mushroom spines (+11.9%) and fewer thin spines (−7.6%). In order to establish the link between decreased DA levels in the absence of astrocyte VMAT2 and early spine maturation, aVMAT2cKO-Thy1EGFP mice were injected with lentiVMAT2 on P25 or chronically treated with L-DOPA from P20 to P40. In line with the previous findings, both in vivo manipulations were sufficient to normalize spine formation and maturation (*n* = 3 mice each group for spine density, *n* = 4 mice each group for spine morphology, mushroom P28 one-way ANOVA *F* = 12.24, *p* = 0.018, thin P28 one way ANOVA *F* = 34.73, *p* = 0.0005, mushroom P40 one way ANOVA *F* = 17.19, *p* = 0.0013, thin P40 one way ANOVA *F* = 13.68, *p* = 0.0026 followed by Tukey’s HSD posthoc test, *p < 0.05; ***p < 0.001; Figs. 4e-i; Supplementary Figures S5m, o). Taken together, these findings are consistent with the proposed role of DA in activity-dependent glutamatergic spine formation during postnatal development [7, 12, 30], and indicate that homeostatic control of DA by astrocytes is necessary for the proper maturation of spines in the developing PFC.

### Decreased DA levels by VMAT2 deletion in astrocytes impairs working memory tasks and behavioural flexibility

Altered dendritic spines density as well as insufficient DA levels in the PFC cause deficits in executive functions [4, 10, 57, 58]. We therefore investigated whether the excision of astrocyte VMAT2 and the consequent reduction in PFC DA levels affects the working memory and cognitive flexibility of aVMAT2cKO mice.

**Fig. 5:**
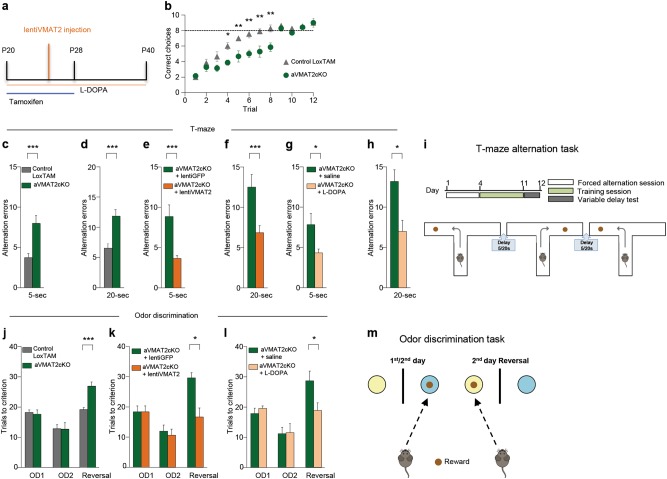
aVMAT2cKO mice have impaired cognitive performance. **a** Event timeline of intra-peritoneal injections of tamoxifen (P20 -P28), local infection with lentiVMAT2 (P25) and intra-peritoneal injection of L-DOPA/benserazide (P20 −P40). **b** T-maze alternation task. The graph shows the number of correct alternations of aVMAT2cKo (green) and control LoxTAM mice (grey) (*n* = 11 in each group, P45-60). The error bars indicate the SEM. **c** and **d** T-maze delayed non-match-to-place (DNMTP). The histograms show the average number of alternation errors made in a T-maze with 5 (**c**) and 20 s of delay (**d**) by aVMAT2cKO (green) and control LoxTAM mice (grey). The error bars indicate the SEM. **e** and **f** Same as in **c** and **d** for aVMAT2cKO mice infected with lentiVMAT2 virus (orange) or control GFP virus (green) (*n* = 6 in each group). Error bars represent SEM. **g** and **h** Same as in **c** and **d** for aVMAT2cKO mice treated with L-DOPA/benserazide (pink) or control saline (green). The error bars indicate the SEM. **i** Representative scheme of T-Maze alternation task. **j** Odour discrimination task. The histograms show the average number of trials needed to reach the criterion (six consecutive correct trails) by recombined aVMAT2cKO and control LoxTAM mice (*n* = 6 per group, P45-60). OD1: discrimination learning; OD2: discrimination repetition; Reversal: reversal of the rule. The error bars indicate the SEM. **k** Same as in (**i**) for aVMAT2cKO mice infected with lentiVMAT2 (orange) or control GFP virus (green) (*n* = 6 per group). The error bars indicate the SEM. **l** Same as in **I** for aVMAT2cKO mice treated with with L-DOPA/benserazide (pink) or control saline (green) (*n* = 6 per group). The error bars indicate the SEM. **m** Representative scheme of odor discrimination task

Two T-maze tasks were used to test working memory (Fig. 5i). In the T-maze alternation task, task acquisition by mice requires short-memory retention [59]. We found that both aVMAT2cKO mice and controls reached the pre-set criterion (correct choices in seven out of eight consecutive trials) within 9 days of training, but the aVMAT2cKO mice required significantly more trials to reach the criterion (*n* = 11 each group, two-way ANOVA *F* = 48.7, *p* = 0.006 followed by Bonferroni’s posthoc correction, **p < 0.01; *p < 0.05; Fig. 5b). In the second test, the T-maze delayed non-match-to-place task [60], each trial is divided into two phases: in the sample phase, one of the two goal locations is blocked by a wall and the mouse is directed towards a food reward in the open location (i.e., the animal must encode the location of the sample goal); in the delay phase, the mouse is returned to the starting box and has to maintain the sample goal in its working memory during a variable delay. While alternating, the aVMAT2cKO mice made many more errors than the control mice using both the 5 and 20 s inter-trial intervals (*n* = 6 each group, Student’s t test, ***p < 0.001; Figs. 5c, d), which suggested a deficit in working memory.

As lentiVMAT2 and L-DOPA were able to restore extracellular levels of DA in the PFC and rescue the functional and morphological defects observed in glutamatergic neurons, we also tested whether such in vivo manipulations could improve the working memory deficiency of aVMAT2cKO mice. In a different set of experiments, aVMAT2cKO mice were injected with a lentivirus expressing VMAT2 or GFP at P25 or chronically treated with L-DOPA (20 mg kg^−1^ i.p.) or vehicle from P20 to P40 (Fig. 5a), and were then given the T-maze delayed non-matching to position (DNMTP) task. As shown in Fig. 5 e–h, working memory significantly improved, thus indicating that normalizing extracellular DA levels by means of the re-expression of astrocyte VMAT2 or treatment with L-DOPA was sufficient to prevent the onset of cognitive deficits (*n* = 6 mice each group, Student’s *t* test, ***p < 0.001; *p < 0.05).

Subsequently, cognitive flexibility was assessed as a reversal learning ability in aVMAT2cKO or control mice respectively injected with a lentiVMAT2 or control lentiGFP virus given a two-choice odour discrimination task. The mice were required to learn to discriminate two odours in order to find a reward and, upon attaining the learning criterion, the odour-reward contingency was reversed on the second training day (Fig. 5m) [61]. Although they were not impaired in acquiring the first odour discrimination, aVMAT2cKO mice injected with lentiGFP required significantly more trials than the control mice to achieve the reversal learning criterion (*n* = 6 each group, Student’s t-test, ***p < 0.001; Fig. 5j), which is consistent with a deficit in acquiring the reversal of the rule. The restoration of astrocyte VMAT2 in early adolescence (P25) reduced the number of trials to control levels (*n* = 6, Student’s *t* test, *p < 0.05; Fig. 5k).

We also evaluated the effect of restoring DA levels in the reversal learning deficit by chronically treating the mice with L-DOPA (20 mg kg^−1^ i.p.) or vehicle from P20 to P40. As shown in Fig. 5l, the increased number of trials required to reach the criterion was significantly rescued in the aVMAT2cKO mice treated with L-DOPA in comparison with those treated with vehicle, thus indicating that the frontal cognitive deficits in the aVMAT2cKO mice were due to a deficiency in DA during the postnatal maturation of PFC circuits (*n* = 6 mice each group, Student’s *t* test, *p < 0.05).

## Discussion

Although the recent RNA sequencing of purified astrocytes from juvenile human and murine brain have revealed the presence of VMAT2 in astrocytes [25], the physiological role of mammaianl astrocytic VMAT2 has never been investigated. We verified the presence of VMAT2 in astrocytes and, using a loss-of-function genetic strategy, examined whether and how a deficit in astrocyte VMAT2 may have an impact on brain physiology. An immunohistochemistry survey of various brain areas revealed that VMAT2 immunolabelling was particularly enriched in the astrocytes of the frontal cortex (including the PFC) from the second postnatal week, but almost absent in other DAergic areas such as the ST and VTA, thus suggesting that astrocytic VMAT2 plays a specific functional role in frontal cortical regions. In line with this, the ubiquitous deletion of astrocyte VMAT2 caused an immediate imbalance of DA homeostasis with a concomitant decrease in the extracellular levels of DA specifically in the PFC. The decreased DA levels and accompanying decrease in tonic DAergic modulation enhanced excitatory transmission, hindered synaptic plasticity, increased the number of mature dendritic spines of L5 pyramidal neurons and affected the development of cognitive processes associated with the PFC which are frequently observed in many developmental psychiatric disorders. The restoration of astrocyte VMAT2 or treatment with L-DOPA in the initial period of DA imbalance was sufficient to prevent the cognitive deficiencies.

Other astroglial genes involved in DAergic metabolism included the plasma membrane low affinity transporter OCT3 and metabolic enzyme MAOB. Indeed, DA homeostasis in the PFC shows particular features as this brain region contains drastically less high-affinity dopamine transporter (DAT) than the ST [62], and several studies have shown that DA uptake through DAT plays a marginal role in clearing extracellular levels of DA in the PFC [63]. In the presence of low concentrations of DAT, mature PFC depends on secondary mechanisms such as the COMT and MAO metabolic enzymes and, possibly, uptake by the norepinephrine transporter in order to clear released DA from extracellular space [10, 64–67]. Although, the regulatory systems mediating the extracellular clearance of DA in the PFC during postnatal development is still largely unknown and our study provide key findings advancing our understanding of such homeostatic control.

First, we found that some of the determinants of DA homeostasis (i.e., OCT3, VMAT2 and MAOB) are expressed in the PFC and enriched in astrocytes during the first 3 weeks of postnatal development. Earlier studies have suggested that low-affinity DA uptake in the PFC may take place through non-cognate transporters [68], and potential candidates that could ensure low-affinity DA transport in the PFC include the organic cation transporter (OCT) family [69–72] and the plasma membrane monoamine transporter (PMAT) [73] . Although they are mainly expressed in neurons, OCT3 and PMAT have been reported in astrocytes of adult rodents [23, 70, 74], where they regulate the extracellular clearance of amines including DA [75] . Both transporters are highly sensitive to inhibition by the isocyanine compound D22. Our data show that the local administration of D22 increases extracellular DA levels in the PFC of juvenile control mice, and completely restores extracellular DA levels in the absence of astrocyte VMAT2, thus indicating that the low-affinity plasma membrane transporter OCT3 may play a particular crucial role in the extracellular clearance of DA especially during postnatal development.

Secondly, we found that the ability of astrocytes to control DA homeostasis depends on the presence of VMAT2. Panel 3d illustrates our hypothesis; in normal condition, DA entering via OCT3 goes into the cytoplasm and into intracellular organelles with a VMAT2-dependent mechanism. Numbers of studies have shown that catecholamine stores exist in a highly dynamic state, with passive outward leakage of catecholamines (including DA) counterbalanced by inward active transport under the control of VMAT2 and that such a leakage from internal stores represents a key determinant of catecholamine metabolism and turnover [76]. The passive leakage of catecholamines from internal stores occurs because the organelles do not exist in a static state but are, instead, often highly dynamics and fusogenics [76]. Thus, in the presence of VMAT2 the ratio between cytosolic and stored catecholamines is kept constant by the activity of degradation enzymes (MAOB or COMT) that works in tandem with VMAT2 to maintain a correct equilibrium between vesicular and free cytosolic catecholamine levels in order to avoid abnormal catecholamine metabolism [77]. In the absence of VMAT2 activity (i.e., deletion of the protein or transport inhibition with reserpine), the passive leakage of catecholamines from internal stores is lost and 100% of catecholamine leaking into cytoplasm is rapidly metabolized by enzymes without a real accumulation in the cytoplasm [77]. Our results suggest that in astrocytes VMAT2 acts in concert with OCT3 to provide effective control of metabolic capacity of astrocytes. Indeed, in the absence of a VMAT2-dependent control of cytosolic levels of extracellular DA taken up by the plasma membrane transporter is promptly metabolized by MAOB without a real accumulation in the cytoplasm [77], thus decreasing the cytosolic concentrations of DA and consequently driving the activity of the plasma membrane transporter. This explanation is fully supported by findings indicating that the level of the MAOB metabolite DOPAC is significantly increased when VMAT2 is pharmacologically inhibited by reserpine (Fig. 3h) or genetically deleted in aVMAT2cKO mice (Fig. 3i). The inhibition of MAOB increases the cytoplasmic levels of DA and restore normal OCT3 uptake, thus increasing extracellular levels of DA to normal levels in aVMAT2cKO mice. Astrocytes, like all eukaryotic cells, contain secretory organelles and express exocytic machinery to promote the fusion of organelles with plasma membrane [78, 79]. Thence, we cannot exclude that the role of astrocytic VMAT2 in the regulation of extracellular levels of DA may also arise from a direct release of DA-containing organelles. It is still widely debated whether and how astrocytes release chemical transmitters in vivo [80], therefore the functional relevance of a direct release of DA from astrocytes in the brain physiology will require further investigations.

Third, we found that relatively modest increase in the metabolism of DA and the concomitant decrease in extracellular DA levels in the PFC in the absence of VMAT2 can have significant effects on cognitive performances thus highlighting the cognitive consequences of postnatal DA deficiency. In line with recent data showing the effect of DA on the activity-dependent formation of spines in juvenile mice [30], we found a close temporal relationship between a significant decrease in PFC DA levels at P25 and accelerated spine maturation from P28 in mice whose astrocyte VMAT2 had been conditionally deleted. In the absence of astroglial VMAT2, recordings of excitatory synapses onto L5 pyramidal neurons showed an increase in the frequency of mEPSCs due to an increased probability of presynaptic release and, consistently, the spines in PFC L5 became larger than those observed in controls, thus indicating that an essential function of astrocytic control of DA homeostasis is to maintain the efficacy of excitatory synapses during PFC development. The effect of the lack of astrocyte-controlled DA metabolism on synaptic strength is consistent with accelerated synaptic development, and suggests that appropriate DA tone acts as a developmental repressor and may account for the neotenic traits of the PFC. Indeed, the development and maturation of cortical areas innervated by DA such as the PFC are slower than that of other cortical areas [81, 82] . This increase in synaptic activation may also be associated with an increase in activity-dependent plasticity mechanisms that promote the spine maturation classically associated with LTP and consequently reduce the dynamic range of further synaptic enhancement. Accordingly, we found that LTP formation was compromised in aVMAT2cKO mice. The essential role of astrocytic VMAT2 in the control of spine formation and maturation, may also arise from the cells’ ability to regulate DA metabolism within the neuropil and thus directly maintain proper dopaminoception [83] . Indeed, an additional possibility is that proper DA levels may directly regulate formation and maturation of spines. This interpretation is supported by the observation that the over-activation of D2 receptors, which are primarily concerned with background DA [84], leads to a significant reduction in spine density [12, 85] . It may therefore be hypothesized that the decrease of DA observed in our aVMAT2cKO mice relieves tonic repression by D2 receptors activation during PFC development, as in the case of the hippocampus [12]. As previously reported [53, 86], the increase in basal excitation appears sufficient to disrupt synaptic plasticity and behaviour. The dysregulation of dendritic spine shape, size and number, and therefore of synaptic structures and functions in the PFC, accompanies a large number neurodevelopmental disorders [1], including autism and schizophrenia [4], and we found that it is concomitant with the appearance of deficits in long-term synaptic plasticity and cognition.

The defect in executive functions observed in VMAT2-deficient mice is explained by hypoDAergic state caused by the lack of control in DA homeostasis by astrocytes. Indeed, when DA levels were restored in the defective astrocytes (by re-expressing VMAT2 in astrocytes or by chronically treating mice with L-DOPA) both the synaptic activity and plasticity and cognitive performance were completely rescued. The fact that the deletion of VMAT2 and subsequent decrease in DA causes excessive developmental neural excitation strongly suggests a neural network that is resistant to experience-dependent refinement, and therefore prone to cognitive and behavioural deficits. Rescuing astroglial VMAT2 and DA levels in the critical postnatal period during which spines are subject to DA regulation [12, 30] corrects the cognitive abnormalities in VMAT2-deficient mice, and therefore suggests that defective synaptic structures and functions developed in the third postnatal week may crucially contribute to dysregulating PFC neural networks and promote the genesis of cognitive deficits.

In conclusion, our findings advance our understanding of the genesis of cognitive impairments reminiscent of psychiatric disorders and provide a new framework that includes astrocytes at the core of the mechanisms underlying the DAergic modulation of executive functions.

## Electronic supplementary material

Supplementary table 1

Supplementary table 1

Supplementary figures legends

Supplementary methods

Supplementary figures legends

Supplementary methods

Supplementary Fig S1a-d

Supplementary Fig S1e-h

Supplementary Fig S1i-j

Supplementary Fig S1k-q

Supplementary Fig S1r

Supplementary Fig S2a-h

Supplementary Fig S2i-s

Supplementary Fig S3a-b

Supplementary Fig S3c-h

Supplementary Fig S4a-f

Supplementary Fig S5a-f

Supplementary Fig S5g-l

Supplementary Fig S3m-o
