# Supplementary material for: Dysfunction of homeostatic control of dopamine by astrocytes in the developing prefrontal cortex leads to cognitive impairments
Source: Mol Psychiatry. 2018 Aug 20;25(4):732–49. doi: 10.1038/s41380-018-0226-y (PMC7156348; doi:10.1038/s41380-018-0226-y)
Supplement: Supplementary file 1 — Supplementary table 1 [file 41380_2018_226_MOESM1_ESM.docx]

**Supplementary Table 1: Antibodies list**

| Antigen | Host | Dilution | Company | References |
| --- | --- | --- | --- | --- |
| GFAP | Mouse | 1:1000 | Chemicon, Millipore (MAB3402) | Ravizza et al., Neuroscience, 2006 |
| GS | Mouse | 1:1000 | Chemicon, Millipore (MAB302) | Shubert et al., Glia, 2011 |
| MAO B | Goat | 1:50 | Santa Cruz (sc-18401) | Jo et al., Nature medicine, 2013 |
| OCT-3 | Rabbit | 1:100 | Alpha diagnostics (OCT31-S) | Cui et al., PNAS, 2009 |
| TH | Rabbit | 1:200-1000 | Millipore (AB152) | Zhang et al., Nature Neuroscience, 2015 |
| VMAT2 | Rabbit | 1:500-1000 | Chemicon, Millipore (AB1767) | Wang et al., Neuron 1997 |
| Cux1 | Rabbit | 1:50 | Santa Cruz (sc-13024) | Fenelon et al., PNAS, 2011 |
| Iba1 | rabbit | 1:500 | WAKO (019-19741) | Kanazaea et al., JBC, 2002 |
| NeuN | Mouse | 1:200 | Millipore (MAB377) | Magavi et al., Let. Nature 2000 |
| VMAT2 | Goat | 1:500 | Synaptic System (138 302) |  |
| HA.11 | mouse | 1:500 | Covance (MMS-101P) |  |
| GLT-1 | Rabbit | 1:250 |  | gift from N.C. Prof. Danbolt |
| GLAST25 | Rabbit | 1:250 |  | gift from N.C. Prof. Danbolt |
| GFP | Rabbit | 1:200 | Millipore (AB3080) | Knott GW et al., Nature Neuroscience 2006 |
| S100β | Mouse | 1:1000 | Sigma (S2532) | Monai et al., Nature communication, 2016 |
| α-tubulin | Mouse | 1:2000 | Santa Cruz (sc-8035) | Zhang et al., 2000, Science |
