# Supplementary material for: Dysfunction of homeostatic control of dopamine by astrocytes in the developing prefrontal cortex leads to cognitive impairments
Source: Mol Psychiatry. 2018 Aug 20;25(4):732–49. doi: 10.1038/s41380-018-0226-y (PMC7156348; doi:10.1038/s41380-018-0226-y)
Supplement: Supplementary file 3 — Supplementary figures legends [file 41380_2018_226_MOESM3_ESM.docx]

**Supplementary Figure S1 (related to Figure 1). Additional data concerning the expression of TH, MAOB, OCT3 and VMAT2 in astrocytes.**

(a) Histograms show qPCR analysis of the relative expression of VMAT2, OCT3, MAOB and TH mRNA in EGFP-positive FACS-sorted astrocytes in comparison with β-actin.

(b) PFC astrocytes were isolated by means of FACS-sorting, and some were re-analyzed to reveal >99.5% purity.

(c) qPCR analysis of the relative expression of GFAP, MOG, Calb1 and Syt1 mRNA in ECFP- and EGFP- positive FACS-sorted astrocytes in comparison with β-actin.

(d) Confocal images showing OCT3 immunolabeling (red) in rat PFC (P30). Astrocytes are stained with GS. Scale bar: 100 μm; high magnification scale bar: 33 μm.

(e) Confocal images showing monoamine oxidase B (MAOB) immunolabelling (green) in rat PFC (P30); astrocytes are stained with GS (red). Scale bar: 100 μm; high magnification scale bar: 24 μm.

(f) Confocal images showing tyrosine hydroxylase (TH) immunolabelling (green) in rat PFC (P30); astrocytes are stained with glutamine synthase (GS, red). Scale bar: 100 μm; high magnification scale bar: 39 μm.

(g) Western blot analysis of whole-brain tissues from post-natal day 30 (P30) hemagglutinin (HA)- tagged VMAT2 mice revealed one HA immunoreactive band of 50 kDa (Covance antibody).  Confocal images showing HA immunolabelling (red) in the frontal cortex (FC) and ventral vegmental area (VTA) of P30 HA-tagged VMAT2 mice; the astrocytes are stained with S100β (green). Scale bars: 25 μm.

(h) Series of electron micrographs showing immunoperoxidase black precipitate for HA in boutons (term, falsely colored orange) and one astrocyte of layer 5 of the PFC (Ast, falsely coloured green). Scale bars: 1 μm.

(i) Confocal sections showing VMAT2 immunolabelling (red) in P7, P14 and P30 mouse prefrontal cortex (PFC). Astrocytes are stained with glutamine synthase (GS, green). The VMAT2 signal is highlighted by white arrows in astrocytes and grey arrows in neuronal fibres; it is easily recognisable in the PFC astrocytes. Scale bars: 30 μm.

(j) Confocal sections showing GS immunolabelling (red) in ALDH1L1-EGFP mouse prefrontal cortex (PFC). Scale bars: 30 μm.

(k) Southern blot of DNA from the three genotypes: the KO lane (-/-) shows the expected mutant band (neomycin cassette), and the lane of the wild-type littermate (+/+) the expected 2.0 Kb band. The heterozygote lane (+/-) shows both the normal and the mutant bands.

(l) Western blot analysis of newborn whole-brain tissues showing one VMAT2 immunoreactive band (75 kDa; Synaptic System antibody) in the wild-type littermate.

(m) Western blot analysis of newborn whole-brain tissues showing twoVMAT2 immunoreactive bands in the wild-type littermate; the most predominant was 75 kDa and the fainter band 55 kDa (Chemicon antibody). No bands are present in the KO lane.

(n) Confocal images showing VMAT2 immunolabelling (red) in VTA of P5 wild-type and full VMAT2 KO (VMAT2 -/-) mice. Scale bars: 20 μm.

(op) Electron microscopy sections showing the immunogold labelling of VMAT2 in layer V of P5 wild-type (S) and full VMAT2 -/- mice (T), respectively. Scale bars: 500 nm.

(q) Histograms showing the average density of VMAT2 immunogold particles in astrocytic processes and axonal boutons in layer V of P5 wild-type and VMAT2-/- mouse PFC. TheVMAT2 immunogold labelling of axonal boutons in the VMAT2-/-tissues is reduced to background levels. Note that VMAT2 immunogold labelling in P5 astrocytes is very low in both the wild-type and VMAT2-/-mice, but the background levels are not significantly different. (*p < 0.05, one-way ANOVA, Bonferroni’s post-test correction). The error bars indicate the SEM.

(r ) Electron microscopy sections showing double immunogold labelling of GS and VMAT2 or GLT1/GLAST and VMAT2 in layer V of P30 wild-type (S) mice, respectively. Scale bars: 500 nm.

**Supplementary figure S2 (related to Figure 2). Generation and characterization of aVMATcKO mice.**

(a) Schematic diagram of the generation of aVMAT2cKO and control mice. We generated inducible knock-out mice in which astrocyte VMAT2 was specifically deleted in a temporally controlled manner. Mice harbouring a tamoxifen(TAM)-inducible cre recombinase transgene driven by the human astrocytic glial fibrillary acidic protein (hGFAP) promoter [Hirrlinger et al., 2006] were crossbred with mice containing cre excisable loxP sequences in the endogenous VMAT2 gene [Narboux-Neme et al., 2011]. The resulting hGFAPcreERT2VMAT2lox/+ mice (F1) were cross-bred with VMAT2lox/lox mice, and their progeny were hGFAPcreERT2VMAT2lox/lox (aVMAT2cKO) mice (F2) and VMAT2lox/lox (control LoxTAM) mice (F2).

(b) TAM treatment timeline. The mice were treated with tamoxifen (TAM) once a day from post-natal day 20 (P20) for eight days. All of the experiments performed between P38 and P45.

(c) Genotyping of aVMAT2cKO mouse and controls. Lane 1: VMAT2 locus with 265 bp bands for the WT allele and 374 bp bands for the lox allele; lane 2: hGFAPcreERT2 transgene at 500 bp.

(d) Histograms showing average weight at the beginning (P20) and end of TAM treatment (P20) in the controls (wt, control LoxTAM, control CreTAM, control CreloxOil) and aVMAT2cKO mice (n=15 each group). The error bars indicate the SEM.

(e) Oxidative damage to DNA (apurinic/apyrimidinic sites) measured in the PFC of control LoxTAM (grey) and recombined aVMAT2cKO mice (green) on P40 (n=5 each group). AP sites: apurinic/apyrimidinic sites. Student’s t test. The error bars indicate the SEM.

(f) TUNEL labelling in recombined aVMAT2cKO and control LoxTAM mice revealed no apoptotic cells (green), which are easily detected in positive control ischemic tissues.

(g) Representative sections showing Nissl staining throughout FC layers of recombined aVMAT2cKO and control (wild-type, LoxTAM, CreTAM, and CreloxOil) mice on post-natal day 40 (P40). The histogram shows the number of Nissl-stained cells per μm2 (n=4 each group). The error bars indicate the SEM. Scale bars: 100 μm.

(h) Representative images showing the co-staining of the pan-neuronal NeuN (green ) and the selective L2/4 marker Cux1 (red) in the FC of recombined aVMAT2cKO and control (wild-type, LoxTAM, CreTAM and control CreloxOil) mice on post-natal day 40 (P40). The histogram shows the number of Nissl-stained cells per μm2 (n=4 each group). The error bars represent SEM. Scale bars: 100 μm.

(i) Representative Western blot of glial fibrillary acidic protein (GFAP) expression in the PFC of aVMAT2cKO and control LoxTAM mice. The histogram shows the quantitative data (normalised to tubulin) indicating no difference between the two groups (n=3 each group). The error bars indicate the SEM.

(j, k) Confocal sections showing TAM-induced recombination in the prefrontal cortex (PFC) and ventral tegmental area (VTA) of CreERT2XROSA(R )26-EYFP mice reporter (P40). EYFP immunolabeling (green) does not co-localize with the neuronal marker NeuN (red). Nuclei are stained with DAPI (bleu). Scale bar 50 μm.

(l-q) FACS analyses of brain homogenates obtained from aVMAT2cKO and control LoxTAM mice previously immunolabelled for NeuN and DAPI. The asterisks indicate the cell population used for PCR analysis.

(r) Purity of immunolabelled FACS sorted cells. NeuN in green and DAPI in blue.

(s) Control of semi-quantitative PCR analysis of DNA isolated from FACS-sorted NeuN+ and NeuN- cells using the β-actin promoter. The standard PCR curve for β-actin promoter shows equal copy numbers between NeuN+ and NeuN- genomic DNA.

**Supplementary Figure S3 (related to Figure 3). Additional data concerning DA uptake, the extracellular DA levels in aVMAT2cKO mice in the presence of OCT3 and MAOB inhibitors and the characterization of lentivirus carrying VMAT2.**

(a) Dopamine uptake in primary astrocytes using [3H]-Dopamine. Curves represent time –dependent accumulation of [3H]-Dopamine (3mM DA and 150 nM of [^3^H]-Dopamine as a tracer) in primary cultured astrocytes (wt) in presence (grey curve) or absence (black curve) of D22 for various period of times (min). The error bars indicate the SEM.

(b) Extracellular levels of DA in the PFC of recombined aVMAT2cKO and control LoxTAM mice: i) chronically treated with L-DOPA/benserazide (20 mg+12 mg i.p. daily) or deprenyl (10 mg/kg i.p daily); ii) locally perfused with 100 μM decynium-22 (1,1′-Diethyl-2,2′-cyanine; D-22); or iii) locally infected with astrocyte-targeted lentiGFP or lentiVMAT2 viruses. Data expressed as percentages of baseline levels (n=4-6; **p<0.001, *p<0.05 one-way ANOVA followed by Tukey’s post hoc test. The error bars indicate the SEM.

(c) Representative Western blot of VMAT2 expression in the PFC of aVMAT2cKO, control LoxTAM (grey), and recombined aVMAT2cKO mice (green) infected with astrocyte-targeted lentiVMAT2 (orange). The data are normalized to tubulin (n=3 per group; *p<0.05 one-way ANOVA followed by post hoc Tukey’s HSD test). The error bars indicate the SEM.

(d and e) Confocal images showing the cell-specific distribution of lentiGFP virus vector in PFC astrocytes. Immunolabeling for GFAP, NeuN and TH is shown in red. Note that GFP (green) is only detectable in GFAP-positive astrocytes (red). Scale bars: 20 μm.

(f) Confocal images showing GFAP expression in PFC of control and lentivirus infected astrocytes (red). Immunolabeling for GFAP is shown in green. Scale bars: 20 μm

(g) Representative Western blot of glial fibrillary acidic protein (GFAP) and ionized calcium-binding adapter molecule 1 (Iba1) expression in the PFC of aVMAT2cKO mice infected with lentiVMAT2 and of control LoxTAM mice (P40). The histogram shows the quantitative data (normalized to tubulin) indicating no difference between the two groups. The error bars indicate the SEM.

(h) Confocal images reconstruction from coronal brain section showing the volume (0.05 mm^3^) and site of injection of lentiGFP virus vector in infralimbic and prelimbic mPFC (IL and PrL). The injections were performed 1.90mm rostral to bregma, 0.2mm lateral to midline and 2.0mm below the skull surface. Scale bars: 100 μm

**Supplementary Figure S4. No alteration in dopaminergic neuronal activity in aVMAT2cKO mice.**

a) Confocal sections showing TH staining (black) in the PFC of P38 recombined aVMAT2cKO and control LoxTAM mice. Scale bar: 100 μm.

b) Western blot analysis of TH expression in PFC tissues from P38 recombined aVMAT2cKO and control LoxTAM mice. The histogram shows the quantification of TH expression in aVMAT2cKO mice expressed as a percentage of that of control LoxVMAT2 (93.6%). The data are normalised to tubulin. The error bars indicate the SEM.

c) Representative VTA-containing coronal slice with the pontamine sky blue dye (red arrow). Below, typical waveform and firing pattern of a putative DA neurons recording in the VTA and schematics of the recording sites in the two experimental groups (control LoxTAM and aVMAT2cKO, P40-45). Right: Histograms showing baseline firing parameters (firing rate, spikes in burst and coefficient of variation) of putative DA neurons in LoxTAM and aVMAT2cKO mice. The error bars indicate the SEM.

d) Top: Diagram showing rAAV5-hysn-ChR2(H134R)-EYFP virus injection into the VTA (coordinates: AP -3.3, ML +0.4, DV -4.4) of P25 recombined aVMAT2cKO and control LoxTAM mice.An optic fibre is inserted through a guide cannula implanted over the VTA to photo activate VTA neurons. The microdialysis probe is inserted in the PFC (coordinates: AP -2.0, ML +0.6, DV -3.0) Bottom: Representative confocal images showing the selective expression of pAAV-hysn-ChR2(H134R)-EYFP virus in tyrosine hydroxylase(TH)-positive neurons of the VTA. Scale bar: 200 μm.

e) High-magnification images showing the co-localisation of EYFP and TH signals. Scale bar: 20 μm.

f) Phasic photo-stimulation of the VTA in recombined cVMAT2cKO and control LoxTAM mice (25 flashes at 50 Hz delivered with a periodicity of 1 min). The histograms show the phasic-stimulated extra-cellular levels of DA calculated in the perfusates of the *in vivo* microdialysis of the PFC in recombined aVMAT2cKO and control LoxTAM mice (P40-45). The error bars indicate the SEM.

**Supplementary Figure S5 (related to Figure 4). Additional data concerning alterations in synaptic activity and plasticity in aVMAT2cKO mice**.

(a) On the left, I/O graph showing the maximum EPSP slope plotted against the stimulus. The extracellular recordings were obtained in aVMAT2cKO and control mice by placing the stimulating electrode at the border of layer I (L1) or layers II/III (L2,3), and the extra-cellular recording electrode in L5 of the PFC. The PFC slices prepared from aVMAT2cKO (n=25 slices/12 mice) mice (green) show enhanced basal synaptic transmission in comparison with control groups (wt, n=12 slices from 4 mice ; LoxTAM, n= 23 slices from 13 mice; CreTAM, n=11 slices from 4 mice; CreLoxOIL, n=17 slices from 7 mice). On the right, I/O graphs showing the afferent volley (AV) slope (top) and the fEPSP/AV (bottom) against stimulus intensities. Deletion of VMAT2 in aVMAT2cKO mice does not change cell excitability. The error bars indicate the SEM. ***p<0.001, two way ANOVA. The example traces show superimposed fEPSP as stimulation intensity increases. Calibration bars: 5 ms, 0.5 mV.

(b) Graph showing the pooled paired-pulse ratios (slope2/slope1 x100 -100) plotted against the interstimuli interval (ISI) in aVMAT2cKO and control groups mice. The deletion of astrocyte VMAT2 led to a clear deficit in short-term facilitation, which indicates an increased probability of release and corroborates enhanced synaptic transmission. The error bars indicate the SEM. ***p<0.001, two-way ANOVA. The example traces show facilitation at 50 ms ISI. Calibration bars: 10ms, 0.5mV.

(c) Administration of tetanic stimulation (6x50 Hz, 2sec) is indicated by an arrow and the baseline by a

dashed line. Representative traces show responses before (dashed line) and 60 minutes after delivery

(bold line). Calibration bars: 5 ms, 0.2 mV. LTP was significantly reduced in the aVMAT2cKO mice (green) 60 min post induction (controls n=13, +54.48 ± 6.55% change; aVMAT2cKO, n=21, +29.21 ± 4.46% change).

(d) Intracellular recording of L5 pyramidal neurons revealed that mEPSC frequency was increased in

the mice lacking astrocyte VMAT2 (lower left panel, green), whereas its amplitude remained unchanged (lower right panel; controls n=7 slices from 4 mice, 1.54 ± 0.51 Hz; aVMAT2cKO: n=13 slices from 2 mice, 3.47 ± 0.57 Hz, *p<0.05). This corroborates the increased probability of release.

(e) Firing activity of L5 pyramidal neurons. Firing activity of layer V pyramidal neurons was induced by square depolarizing current pulses in whole-cell current-clamp recordings. Left, examples of firing activities recorded in aVMAT2cKO (n=22 slices from 5 mice, green) and in control groups mice (wt, n=12 slices from 4 mice ; LoxTAM, n= 18 slices from 8 mice; CreTAM, n=7 slices from 2 mice; CreLoxOIL, n=22 slices from 6 mice). Right, graph showing the firing frequency plotted against injected current. Invalidation of VMAT2 in astrocytes does not affect neuronal excitability.

(f) Neuronal membrane properties are unchanged in aVMAT2cKO mice, indicating that the membrane integrity and current spread are unaffected by deletion of VMAT2 in astrocytes.

g) Effect of DA (100 µM) on aVMATcKO mice. Left panel shows the time-course of the inhibitory effect of DA on fEPSP (-36.01±7.07% vs baseline, n= 8 slices from 3 mice). Middle panel represents fEPSP slope against stimulus intensities normalized to the maximal value obtained before (green circles) DA application (green squares, n=6 slices from 3 mice). Right panel displays the rescuing effect of DA on LTP (+47.88±11.52% vs baseline, n=6 slices from 3 mice). The error bars indicate the SEM. ***p<0.001, two way ANOVA.

h) Effect of sulpiride (50 µM) on WT mice. Left panel shows the time-course of the potentiating effect of sulpiride on fEPSP (+32.88±11.71% vs baseline, n= 8 slices from 4 mice). Middle panel represents fEPSP slope against stimulus intensities normalized to the maximal value obtained before (black circles) sulpiride application (black diamonds, n=5 slices from 3 mice). Right panel displays the down regulatory effect of sulpiride on LTP (+20.32±13.24%, vs baseline, n=4 slices from 3 mice). The error bars indicate the SEM. ***p<0.001, two way ANOVA.

i) Effect of sulpiride (50 µM) on LoxTAM mice. Left panel shows the time-course of the potentiating effect of sulpiride on fEPSP (+27.32±12.04% vs baseline, n= 6 slices from 3 mice). Middle panel represents fEPSP slope against stimulus intensities normalized to the maximal value obtained before (gray circles) sulpiride application (gray diamonds, n=6 slices from 3 mice). Right panel displays the down regulatory effect of sulpiride on LTP (+19.41±12.43%, vs baseline, n=6 slices from 3 mice). The error bars indicate the SEM. ***p<0.001, two way ANOVA.

j) Effect of sulpiride (50 µM) on aVMAT2cKO+lentiVMAT2 mice. Left panel shows the time-course of the potentiating effect of sulpiride on fEPSP (+30.54±6.30% vs baseline, n= 6 slices from 3 mice). Middle panel represents fEPSP slope against stimulus intensities normalized to the maximal value obtained before (orange circles) sulpiride application (orange diamonds, n=6 slices from 3 mice). Right panel displays the down regulatory effect of sulpiride on LTP (+21.39±14.33%, vs baseline, n=8 slices from 3 mice). The error bars indicate the SEM. ***p<0.001, two way ANOVA.

k) Effect of sulpiride (50 µM) on aVMAT2cKO+L-DOPA mice. Left panel shows the time-course of the potentiating effect of sulpiride on fEPSP (+34.38±15.22% vs baseline, n= 10 slices from 4 mice). Middle panel represents fEPSP slope against stimulus intensities normalized to the maximal value obtained before (pink circles) sulpiride application (pink diamonds, n=8 slices from 4 mice). Right panel displays the down regulatory effect of sulpiride on LTP (+21.10±12.28%, vs baseline, n=6 slices from 4 mice). The error bars indicate the SEM. ***p<0.001, two way ANOVA.

l) Histogram showing the effect of DA and sulpiride on the pooled paired-pulse ratios (slope2/slope1 x100 -100) for the 50 msec interstimuli interval (ISI) in the different mice groups. DA rescues paired pulse ratios in aVMAT2cKO mice while sulpiride impairs this short-term plasticity in wt, control LoxTAM, aVMAT2cKO+virus and aVMAT2cKO+L-DOPA mice.

(m-o) Histograms showing the percentage of filipodia and stubby spines in control LoxTAM (grey), recombined aVMAT2cKO (green), recombined aVMAT2cKO+L-Dopa/Benserazide (pink) and recombined aVMAT2KO+lentiVMAT2 mice (orange) at three developmental stages (P20, P28 and P40): n=4 for each group. Student t test (P20) and one-way ANOVA followed by post hoc Tukey’s HSD (P28 and P40). *p<0.05; **p<0.001. The error bars indicate the SEM.
