# Supplementary material for: Dysfunction of homeostatic control of dopamine by astrocytes in the developing prefrontal cortex leads to cognitive impairments
Source: Mol Psychiatry. 2018 Aug 20;25(4):732–49. doi: 10.1038/s41380-018-0226-y (PMC7156348; doi:10.1038/s41380-018-0226-y)
Supplement: Supplementary file 4 — Supplementary methods [file 41380_2018_226_MOESM4_ESM.docx]

# Supplementary Materials and methods

**Maintenance, breeding and genotyping**. All animal studies were approved by the « Service de la consommation et des affaires vétérinaries du Canton Vaud ». Mice and rats were group housed with littermates in standard housing on a 12:12 h light/dark cycle. hGFAPcreE^RT2 1^ comes from Frank Kirchhoff (Molecular Physiology, University of Saarland,Germany), VMAT2lox/lox ^2^ from Bruno Giros (Douglas Mental Health University Institute, Canada), VMAT2BAC-HA3, VMAT2KO-/- (R.H. Edwards, University of California San Francisco, US), ^3^ Thy1-EGFP from Joshua R. Sanes (Harvard University, USA),^4^ tdtomato lox/lox (AI14, Jackson Lab) and ALDH1L1-EGFP (A. Volterra, University of Lausanne, Switzerland). Mice used were C57BL/6 background. Sprague Dawley rats comes from Janvier labs (France). All animals used in these studies were male except for immunohistochemistry analysis. The hGFAPcre^ERT2^ sequence was identified from phalange biopsies using the following primers: 5’- CAGGTTGGAGAGGAGACGCATCA-3’, 5’-CGTTGCATCGACCGGTAATGCAGGC-3’. The VMAT2lox/lox sequence was identified with the following primers: 5’-GACTAGGGACAGCACAAATCTCC-3’, 5’- GAAACATGAAGGACAACTGGGACCC-3’. ROSA26-eYFP-hGFAPcre^ERT2 5^ have been obtained from Frank Kirchhoff and mice were C57BL/6 background. The ROSA26-EYFP sequence was identified with the following primers: 5’-AAAGTCGCTCTGAGTTGTTAT-3’, 5’-GCGAAGAGTTTGTCCTCAACC-3’, 5’-

GGAGCGGGAGAAATGGATATG-3. PCR reaction product coupled with syber green migrates in a 1,5% agarose gel then bands are revealed by UV lights.

**FACS of neurons and semi-quantitative PCR**. Whole brains were dissected from P40 mice and samples were prepared as described previously. ^6^ The sorted cells were collected at 10,000 r.p.m. for 20 min and genomic DNA was prepared from NeuN+ and NeuN- fractions using Wizard Genomic DNA Purification Kit (Promega). Genomic PCR for VMAT2 gene exon 2 and neomycin box with two couples of primers: WTForward (5'-CATCGTGTTCCTCGCGCTGC-3’) and WTReverse (5'- GGGATGCTGTCACCTGGG- 3') for exon1 and NeoForward (5'-CCGCTCCCGATTCGCAGCG-3') and NeoRev (5'-GCAGCAGCTTAGCACACTGG-3’) for neomycin box. For genomic PCR of α-actin promoter the following oligonucleotide were used: forward 5’-CCCAACACACCTAGCAAATTAGAACCAC-3’and reverse 5’-CCTGGATTGAATGGACAGAGAGTCACT-3’. PCR reaction product coupled with syber green migrates in a 1,5% agarose gel then bands are revealed by UV lights. No experiments were discarded.

**Cell culture**. Enriched astrocytic cultures were prepared from cortices of 0- to 2- day C57BL/6, LoxTAM and aVMAT2cKO pups, as previously described.^7,8^ Briefly, dissociated neural cells were initially plated into 25m^2^ flasks and maintained in minimum essential medium (MEM, Gibco, 21090-

022) supplemented with Fetal Bovine Serum (FBS, 10%, PAA cell culture company, A15-101), L- glutamine (2mM, Gibco, 25030-024), D-glucose (20mM), penicillin/streptomycin (100 U/ml,

100µg/ml, Gibco, 15140-22) and kept at 37°C in humidified 5%CO_2_/95% air. After 14-18 days in culture, cells were purified for astrocytes (>99%) using an orbital shaker ^9^. No experiments were discarded.

**FACS of astrocytes**. Mice from GFAP-ECFP transgenic litter (TgN(hGFAP-ECFP),^10^ cre^ERT2^XVMAT2XR26-tdtomato, cre^ERT2^XR26-tdtomato and ALDH1L1-EGFP were used. The frontal part of the brain was isolated and the olfactory lobes were removed by crude dissection. The tissue was diced with a curved-blade surgical scalpel. To make a suspension of single cells, the tissue was enzymatically dissociated via the incubation at 33°C for 80 minutes in 10ml of papain solution (20U/ml, Sigma- Aldrich, P3125). The solution was prepared in dissociation buffer completed with EDTA (0.5mM, pH=8), L-cysteine-HCL (necessary to activate the papain, 1mM, Sigma-Aldrich, C7477) and DNase (125U/ml, Sigma-Aldrich, D4527). Dissociation buffer contained Earle’s balanced salt (EBSS, Sigma- Aldrich, E7510), D(+)-glucose (22.5mM), NaHCO3 (26mM) and requires equilibration with 5% CO2 and 95% O2 gas before use and during papain treatment. ^11^ After papain treatment the tissue was washed with 3 x 3 ml dissociation buffer containing BSA (1mg/ml, Sigma-Aldrich, A9647), ovomucoid (also known as Trypsin inhibitor, 1mg/ml, Sigma-Aldrich, T9253) and DNase (125U/ml) (inhibitor solution) and then mechanically dissociated by gentle sequential trituration using a 1mL pipette with 4 x 3 ml fresh inhibitor solution to yield a suspension of single cells. Dissociated cells were layered on top of 12ml of concentrated inhibitor solution (5mg/ml BSA, 5mg/ml ovomucoid, 125U/ml DNase) and harvested by centrifugation (140 x g for 5min). ^11^ Cells were counted and resuspended in a solution containing PBS 1% (phosphate-buffered saline), 0.1% BSA, EDTA (0.5mM) and 4',6- diamidino-2-phenylindole (DAPI, 1µg/ml, Invitrogen, D1306) for FACS purification. CFP or tdtomato positive astrocytes were purified by fluorescence activated cell sorting (FACS) using a MoFlo AstriosEQ High speed cell sorter (Beckman Coulter Life Sciences). Dead cells were gated out using high DAPI staining and forward light scatter. Astrocytes were identified based on high CFP, GFP and tdtomato fluorescence and size. No experiments were discarded.

**Semi-quantitative RT-PCR**. Total RNA from sorted cells was isolated with RNeasy Mini Kit (Qiagen, 74104) and RNA concentration was determined using a NanoDrop 1000 spectrophotometer (Witec AG, Switzerland). Reverse transcription was performed with 600ng of DNase-treated total RNA using M-MLV reverse transcriptase (Promega, M3683). The quantitative real-time PCR was done on C1000T Thermal Cycler (CFX96 real-time PCR system, Bio-Rad) using SYBR Select Master Mix for CFX (Applied Biosystems – Life Technologies).^9^ The mRNA levels were normalized to the levels of β-actin. The primer sequences used were as follows: GFAP forward 5’-ACCGCATCACCATTCCTGTAC-3’, GFAP reverse 5’-TGGCCTTCTGACACGGATTT-3’; Syt1 forward 5’- GCTTTGAAGTTCCGTTCGAG-3’, Syt1 reverse 5’-AGCATGTCTGACCAGTGTCG-3’; MOG forward 5’-CCTGGTTGCCTTGATCATCTGCTAC-3’, MOG reverse 5’-TCTACTCGGTATCCAGAATGTGTCTG-3’; VMAT2 forward 5’-GCGAGCATCTCTTATCTCATTGG-3’, VMAT2 reverse 5’-AAATGCTGATCCCAACAACTATCA-3’; OCT3 forward 5’-CTATGCAGCGGACAGATATGG-3’, OCT3 reverse 5’-AGCGGAAAATCACAAACACAGAA- 3’; MAOB forward 5’-ACTCGTGTGCCTTTGGGTTCAG-3’, MAOB reverse 5’-TGCTCCTCACACCAGTTCTTCTC-3’; TH forward 5’-GAAGGGCCTCTATGCTACCCA-3’, TH reverse 5’-TGGGCGCTGGATACGAGA-3’; Calb1 forward 5'-GCT GCA GAA CTT GAT CCA GGA-3'; Calb1 reverse 5'-TCC GGT GAT AGC TCC AAT CC-3'; ALDH1L1 forward 5’-CATCTTTGCTGACTGTGACCTC-3’; ALDH1L1 reverse 5’-TTCACACCACGTTGGCAATAC-3’; GLAST forward 5’-CGCGGTGATAATGTGGTATGC-3’; GLAST reverse 5’-CAAGCTGTCCCCCAATCACA-3’; β-actin forward 5’-GGCTGTATTCCCCTCCATCG-3’, β-actin reverse 5’-CCAGTTGGTAACAATGCCATGT-3’. For quantification ΔΔCT method was used. No experiments were discarded.

**Time course of dopamine uptake in primary astrocytes**. Primary astrocytes isolated from C57BL/6, LoxTAM and aVMAT2cKO pups were seeded in a 96-well plate in the incubator at 37 °C and 5% CO_2._ At the day of the experiment, cells were washed twice with Krebs Ringer Hepes (KRH) buffer (125mM NaCl, 25 mM hepes, 5.6 mM glucose, 4.8 mM KCl, 1.2 mM KH2PO4, 1.2 mM CaCl2, 1.2 mM MgSO4, pH 7.4) and then preincubated for 60 min at 37 °C in KRH buffer in the presence of reserpine (1 µM) or reserpine plus the MAOB inhibitor deprenyl (1 µM, Sigma-Aldrich) or vehicle. Afterwards, the cells were incubated for various periods of time (2.5, 5, 10, 20, 40, 50 and 60 min) with 3 mM of dopamine using 150 nM of [3H]-Dopamine (Dihydroxyphenylethylamine 3,4-[ring-2,5,6-3H], 60 Ci/mmol) as a tracer. For chronic effects of VMAT2 deletion on DA uptake, astrocyte cultures of LoxTAM and aVMATcKO were incubated with 4-hydroxytamoxifen (1 µM, daily) (Sigma-Aldrich) in medium for 8 days. After the cells were then incubated with dopamine (3 mM) using 150 nM of [3H]-Dopamine in presence or absence of deprenyl (1 µM) in KRH buffer (pH 7.4) at 37 °C for 40 min. To stop the reaction, cells were rinsed twice with ice-cold buffer and solubilized with 0.2 N NaOH and 1% SDS. The solubilized samples were transferred into scintillation tubes and added with 3 mL of Ultima Gold scintillation liquid (PerkinElmer Life Sciences). Radioactivity was measured using the Packard Tri-Carb 2100 TR scintillation counter (PerkinElmer Life Sciences) and results expressed as dopamine uptake per 106 of cells. The data were normalized subtracting the non-specific signal obtained by preincubating astrocytes with 100 µM of the OCT3 inhibitor D22 (residual radioactivity was constant at every time point). Data were collected from 6 animals and performed in triplicate. No experiments were discarded.

**Quantification of DOPAC and DA in primary astrocytes**. Primary astrocytes isolated from C57BL/6, LoxTAM and aVMAT2cKO pups were prepared as described previously. Briefly, cells were plated at

0.02-0.03*10^6^ cells per well in 96-well plate. After reaching the confluence (at least 3 days), the cells were pre-incubated with reserpine (1µM for 1h) or deprenyl (1µM for 15 min) in Krebs Ringer Hepes (KRH) buffer (125mM NaCl, 25 mM hepes, 5.6 mM glucose, 4.8 mM KCl, 1.2 mM KH2PO4, 1.2 mM CaCl2, 1.2 mM MgSO4, pH 7.4). Cells were then incubated with dopamine (3 mM) in presence or in absence of reserpine (1µM) or deprenyl (1µM) in KRH buffer (pH 7.4) at 37 °C for various periods of time (10, 20, 40, 50 and 60 min). For chronic effects of VMAT2 deletion on DA and DOPAC metabolism, astrocyte cultures of LoxTAM and aVMATcKO were incubated with 4-hydroxytamoxifen (1 µM, daily) (Sigma-Aldrich) for 8 days. Cells were then incubated with dopamine (3 mM) in presence or absence of deprenyl (1 µM) in KRH buffer (pH 7.4) at 37 °C for 40 min. Finally, after the incubation, the culture medium was removed and the cells were detached and centrifuged for 30 min at 14.000g in centrifugal filter units (Millipore). The intracellular levels of DOPAC and DA were determined in 20 µl of resulting supernatant fluid and the pellet was used for protein determination. The intracellular levels of DOPAC and DA were measured using HPLC with electrochemical detector as described above. No experiments were discarded.

**Tamoxifen treatments in vivo**. The preparation of Tamoxifen (TAM, Sigma-Aldrich) and protocol for treatment was carried out at P20 as described previously.^6^

**Virus preparation**. Self-inactivated (SIN) lentiviruses contain the central polypurine tract (cPPT) sequence, the mouse phosphoglycerate kinase I promoter (PGK), the woodchuck post-regulatory element (WPRE) sequence and the target sequence of miR124 as previously describe.^12^ A Gateway system (Invitrogen) has been used to clone the mouse VMAT2 and GFP cDNA, into the pENTR-D- TOPO plasmid (Invitrogen) and perform an LR clones reaction to transfer into the destination lentiviral vector SIN-cPPT-PGK-Gateway-WPRE-miR124T plasmid. Viruses (lentiGFP, lentiVMAT2) were produced in 293T cells using a four-plasmid system as previously described 13 and were pseudotyped with the G protein of the mokola lyssaviruses^12^.

**Stereotaxic intracranial injections**. Mice (P25) were anesthetized using isoflurane at 5% (w/v), placed in a small animal stereotaxic frame (David Kopf Instruments) and maintained at 2.5% isoflurane (w/c) for the duration of surgery. Corneal and pinch reflexes were regularly tested to confirm anaesthetic depth. Lacryvisc (Aicon, Switzerland) was applied to prevent corneal drying and lidocaine applied topically to the skin overlying the skull. After exposing the skull under aseptic conditions, a small holes were drilled in the skull overlying the prefrontal cortex (AP + 2.0 mm, L ± 0.2 mm and DV -2.0mm).^13^ LentiVMAT2 and lentiGFP were injected (1 μl total volume for site) bilaterally through Hamilton syringe at a rate of 100 nl min-1 using CMA400 Pump (CMA System). For optogenetic experiments, a small holes were drilled in the skull overlying the Ventral Tegmental Area (coordinates: AP -3.3, ML +0.4, DV -4.4mm). Adeno-associated viruses rAAV2-hsyn-ChR2-EYFP was injected (0.5 μL total volume for site of 100 ng p21 per μL) in control LoxTAM and aVMAT2cKO mice unilaterally through Hamilton syringe at a rate of 100 nL min-1 using CMA400 Pump (CMA System). After surgical procedures, mice were returned to their home cage at least for 2 weeks to allow for a max gene expression.

**Western blot analysis**. Mice were killed by decapitation, and brain areas immediately isolated and homogenized on lysis buffer containing 20 mmol/L HEPES, pH 7.4, 10 mmol/L NaCl, 3 mmol/L MgCl2,

2.5 mmol/L EGTA, 0.1 mmol/L dithiothreitol, 50 mmol/L NaF, 1 mmol/L Na3VO4, 1% Triton X-100, and a protease inhibitor cocktail (Roche). Lysate were boiled for 5 min and separated on a denaturating 9% acrilamide gel.^3^ The following primary antibodies were used: VMAT2 (Synaptic System, 1:500), TH (Millipore, 1:1000),^14^ GFAP (Chemicon, 1:1000),^15^ Iba1 (WAKO, 1:500)^16^ and α- Tubulin (Santa Cruz, 1:2000).^17^ The following secondary were used: goat-anti-rabbit, goat-anti-mouse, donkey-anti-goat coupled with IRdye 800 or IRdye 680 (LiCor, Lincoln, 1:10.000) in 30%. Protein bands were revealed by the Odyssey infrared image system (LiCor). No experiments were discarded.

**Tissue preparation, immunohistochemistry and histology**. Sprague Dawley rat, aVMAT2cKO, control LoxTAM, ALDH1L1-EGFP and VMAT2XR26EYFPlox/lox-hGFAPcre^ERT2^ were deeply anesthetized with sodium pentobarbitone (6mg/100g body wt, i.p.) and immediately perfused intracardiacally with fresh 4% paraformaldehyde in 0.1 M phosphate-buffered saline (pH 7.4) . Brains were postfixed overnight, and then equilibrated in 30% sucrose overnight a 4º°C. Sagittal sections (30 um) were cut at -20ºC using a cryostat (Leica) and stored at -80ºC. Sections were permeabilized for 45 min in phosphate-buffered saline containing 0.3% Triton X-100, and 15% donkey or goat serum and then immunolabeled overnight at 4°C using the following primary antibodies: rabbit-VMAT2 (Synaptic System, 1:5600; Chemicon, 1:1000),^18^ mouse-GS (Chemicon, 1:1000), ^19^ rabbit-GFP (Chemicon, 1:200),^20^ rabbit-TH (Millipore, 1:200),^14^ mouse rabbit-GFAP (Chemicon 1:1000),^15^ mouse-NeuN (Millipore 1:200),^21^ rabbit-OCT3 (Alpha Diagnostics, 1:100),^22^ rabbit-Cux1 (Santa Cruz, 1:50), ^23^ mouse-HA.11 (Covance, MMS-101P 1:500), mouse-S100β (Sigma, 1:1000) ^24^ and goat-MAOB (Santa Cruz, 1:10050), ^25^ (Supplementary Table 1). VMAT2 antibody specificity was confirmed by immunolabeling (Figures 2d and e; Supplementary Figure S1f and j) and western blot (Supplementary Figure S1g-i). The day after incubation with primary antibodies, the brain sections were washed again three times in PBS for 10 min and incubated for 1.5h at RT with fluorescent secondary antibodies (AlexaFluor, Invitrogen, Molecular probes, Eugene, Oregon, goat anti-mouse 488, 555, and 633; goat anti-rabbit 488, 555, and 633; 1:300 and 1:400) diluted in PBS. Finally, nuclei were counterstained with 4’, 6-diamidino-2-phenylindole (DAPI) (Invitrogen, Molecular Probes, Eugene, Oregon, 1:10000) and then washed before mounting with a reagent FluorSave (Calbiochem). All images were collected on a Leica confocal imaging system (TCS SP5) with a 40× (1.4 NA) or with 63x (1.4 NA) oil immersion objectives. Sections were acquired every 0.4-μm thickness, and confocal images were analysed using Imaris 7.6.3 (Bitplane AG, Zurich, Switzerland) or Adobe Photoshop CS5 (Adobe System Incorporeted, San José, California, U.S) softwares. VMAT2 signal was identified in astrocytes as follows: astrocytes have been recognized by GS staining. Cells with a clear GS staining were then assessed for VMAT2 expression by analysing the VMAT2 signal inside the GS staining. We considered VMAT2-positive astrocytes when the intensity of VMAT2 signal (a.u.) inside of the GS staining was > 2 fold of background signal calculated in the neuropil. To determinate site and volume of infection with lentiGFP virus, we acquire images using Leica confocal imaging system (TCS SP5) with a 20× (2.5 NA). All images were captured under identical conditions from 1.8mm to 2.3mm AP of infection site (coordinates AP+1.9, L+0.2, V-2.0 mm). Using Blender software, a spheroid was superimposed on top of the image corresponding to the centre of injection, with x, y and z axis following the maximum spread of the fluorescence signal (x = 0.49 mm, y = 0.46 mm, z = 0.48 mm). The volume of the spheroid was calculated using the Neuromorph addons, available for blender ^26,27^. No experiments were discarded.

**TUNEL assays**. Apoptotic cells in PFC cortex tissue was assessed by TUNEL (Dead End^TM^ fluorimetric TUNEL system, Promega) assay. Briefly, brain slides of control LoxTAM, aVMAT2cKO and positive control mice (ischemic brain tissue) were washed with 1% PBS three times and treated with 0.2% triton X-100 in PBS for 10 min. After additional washes, coronal sections were treated with the TUNEL enzyme solution and incubated for 1 h in dark at 37 °C. Finally, nuclei were counterstained with 4’, 6-diamidino-2-phenylindole (DAPI) (Invitrogen, Molecular Probes, Eugene, Oregon, 1:10000) and then washed before mounting with a reagent FluorSave (Calbiochem). All images were collected on a Leica confocal imaging system (TCS SP5) with a 40× (1.4 NA) oil immersion objective. Sections were acquired every 0.4-μm thickness, and confocal images were analysed using Imaris 7.6.3 (Bitplane AG, Zurich, Switzerland) or Adobe Photoshop CS5 (Adobe System Incorporeted, San José, California, U.S) softwares. No experiments were discarded.

**Measurements of DNA Oxidation Levels**: At P40 prefrontal cortex were collected, and DNA was extracted with ethanol precipitation. DNA concentration for each sample were adjusted to 0.1 μg/ml, and numbers of apurinic/apyrimidinic (AP) sites were determined using the DNA Damage Quantification Kit (Dojindo, Rockville, MD) and performed according to the manufacturer’s instructions and as previously described ^28^. No experiments were discarded.

**MAO and COMT enzymatic activity assays**: mice were anesthetized and the prefrontal cortex was quickly excised from the entire brain and submerged in ice-cold solution. The fresh tissues from each mouse were homogenized, and large debris was removed by weak centrifugation. Next, the supernatant was collected and centrifuged at 13,000 r.p.m. for 20 min to obtain a mitochondria-rich fraction. The pellet was resuspended in phosphate buffer and 20 μg were used in each well to determine the activity of MAOA, MAOB and COMT. Enzymatic activity of MAOA or MAOB was measured using an Amplex Red Monoamine oxidase Assay Kit (Molecular Probes) according to the manufacturer’s instructions. Enzymatic activity of COMT was measured using an eEnzymatic assay of Catechol o-methyl Transferase (Sigma-Aldrich EC 2.1.1.6) according to the manufacturer’s instructions. No experiments were discarded.

**Electron Microscopy**. The preparation of the material used to make a VMAT2-HA staining and morphometric analysis of astrocytes and adjacent structures was carried out as described previously ^20,29^ and is briefly outlined below. Mice were transcardially perfused with with 300 ml of 0.2 % glutaraldehyde and 2 % paraformaldehyde in 0.1M PB, pH7.4. Then 60-um sections were cut coronally to the prefrontal cortex, parallel to the imaging window. Sections containing layer V (L5) of prefrontal cortex (PFC) were identified then with a stereomicroscope. After washing and cryoprotection, sections were freeze-thawed in liquid nitrogen and then incubated overnight in primary antibody (mouse-HA.11, Covance, MMS-101P, 1:500). The following day, they were then incubated in biotinylated secondary antibody, followed by avidin biotin peroxidase complex (ABC Elite, Vector Laboratories). This labeling was revealed with DAB and hydrogen peroxide. Sections were then further stained with osmium tetroxide and uranyl acetate, dehydrated, and embedded in Durcapan resin (Fluka). Once the L5 PFC had been located in the resin-embedded section, serial sections (200–300) were cut at 60-nm thickness and collected onto pioloform membrane on single-slot grids. Serial images of the labeled structures were then collected with a digital camera (MegaView III, SIS) inside a Phillips CM12 transmission electron microscope, at a filament voltage of 80 kV. Some labeled astrocytes (n=5), including all of its asymmetric synapses and/or VMAT2 - or dopamine-stained boutons, were subsequently reconstructed in 3D from the serial EM images. Serial micrographs were aligned using Photoshop software (Adobe), and measurements made using the Neurolucida software (Microbrightfield). No experiments were discarded.

**Immunohistochemistry procedure for peroxidase staining**. Each animal (mouse and rat) was transcardially perfused with 300 ml of 0.2 % glutaraldehyde and 2 % paraformaldehyde in 0.1M PB, pH7.4 at room temperature. One hour after the perfusion was stopped, the brain was removed and 60 µm vibratome (Leica VT100) sections cut coronally to the frontal cortex. These sections were then cryoprotected in 2 % glycerol and 20 % DMSO in 0.1 M PBS, for 15 minutes, and freeze-thawed twice in liquid nitrogen prior to overnight incubation in primary antibody (mouse-HA.11, Covance, MMS- 101P, 1:500) in PBS at 4°C. After washing in PBS the sections were then incubated for 2 hours at room temperature in biotinylated secondary antibody (1:500 goat anti-mouse (F)ab fragment, Jackson Laboratories, USA). To reveal this labeling, avidin biotin peroxidase complex (ABC Elite, Vector Laboratories, US) was used for 1 hour, and incubated in 3, 3’-diaminobenzidine tetrachloride (Fluka, Switzerland) and 0.015 % hydrogen peroxide. Following enhancement, the sections were then washed in cacodylate buffer (0.1M, pH7.4, postfixed in osmium tetroxide for 30 mins, followed by 5 mins in uranyl acetate in water, and then dehydrated and embedded in Durcapan resin (Fluka). Once cured in a 65°C oven for 48 hours, the sections were viewed under a light microscope. An image of the blood vessels, taken on the final day of in vivo imaging, was used to compare with the first sections cut from the surface of the cortex, in which the same vessels could be seen. This vessel map was used to locate the dendrites of the imaged cell. No experiments were discarded.

**Lowycril embedding and postembedding immunogold**. Immunogold cytochemistry was carried out as described^7^ using PFC specimens from adult Wistar rats and mice fixed by perfusion through the heart (4% formaldehyde and 0.1% glutaraldehyde). Brain sections were cryoprotected in glycerol, frozen in liquid propane, freeze-substituted with methanol, and embedded in Lowicryl HM20 (Lowi, Waldkraiburg, Switzerland). Ultrathin sections of 80 nm were cut from the blocks obtained. The ultrathin sections were processed with the antibodies according to an immunogold procedure previously described elsewhere.^29^ The sections from Lowicryl-embedded sections were both single labelled with antibodies to rabbit anti-VMAT2 (Synaptic Systems, 1:500), overnight at 4°C. The day after, samples were washed according to the protocol in ^29^ and incubated for 2 h at RT with secondary antibodies coupled to 10nm gold particles (Aurion, Netherlands, donkey anti rabbit, 1:200) or 15nm gold particles (Aurion, Netherlands, donkey anti goat, 1:200). In a double immunogold experiments, astrocytic processes were identified by the presence of filaments and/or by labeling for GLT/GLAST. A mixture of rabbit antibodies to GLT and GLAST25 (both gifts from N.C. Danbolt, Anatomical Institute, University of Oslo) was used at a final dilution of 1 ug/ml. No experiments were discarded.

**Quantitation of brain monoamines**. Monoamines and metabolites were quantified as described previously ^30^ with some modifications. PFC tissue samples were weighted and homogenized with a micro homogenizer in 0.2 ml of ice-cold 0.1N perchloric acid containing 3-ethoxy-4- hydroxyphenylethanolamine oxalate (EHPEA) as internal standard because it is not present in biological fluids and tissues and did not co-elute with NE, DA, DOPAC, 5HT, 5HIAA and HVA. The homogenate was sonicated in this solution at 4°C and centrifuged at 18620 g for 1 min at 4°C and, then filtered through a generated cellulose filter (Millipore, Amicon Ultra 0.5mmL 3K, Ref.:UFC500396) at 18620 g for 45 min. The pellet was used to determine protein concentration with a protein assay kit (Bio-Rad). A calibrator that undergoes the whole extraction protocol (in absence of tissue) was also used to standardize concentrations of bioamines. Perfusates were collected in polypropylene tubes since we observed a significant loss of 5 HIAA and 5HT in glass tubes. NE, DA, DOPAC, 5HT stock solutions were prepared in 0.1N perchloric acid and were stored 2 months at 4°C with no significant degradations. These solutions were further diluted to give a working calibrator solution set at 50 ng/ml concentration. An ESA Coularray Detector (Model 5600A; ESA Inc., Chelmsford, MA) was used for electrochemical monoamine detection. The detector was connected to a high-sensitivity Analytical Cell (model 5011A; Ref. 70-5561, ESA Inc.). The Conditioning Cell (Model 5021A; 70-6068, ESA Inc.) potential was set at +150 mV and the Analytical Cell at +250 mV and -350mV. The mobile phase (1 liter) consisted 9.3 mg EDTA, 398 mg octane sulfonic acid, 9 g dihydrogenosodium phosphate, 100 µl triethylamine, 100 ml acetonitrile with pH adjusted to 3.0 using 85 % phosphoric acid. Fifty microliters of eluate were injected onto the HPLC and the column employed was (RECIPE Analytical Column for Catecholamines in Plasma, ref: 1030) at 25°C. The flow rate was 0.9 ml/min and the retention times were determined for each run. Retention times were as follows: NE, approximately 2.19 min; DOPAC, approximately 3.16 min; DA, approximately 3.48 min; HVA, approximately 6.11 min; and 5HT, approximately 7.33 min. The limits of detections were 0.2 ng/ml for NE, 0.4 ng/ml for DA and DOPAC, and 5-HT. The content in the perfusate was determined by comparison of the area-under the-curve values with those obtained with standards, which were run with each experiment and normalized by protein content. No experiments were discarded.

**In vivo microdialysis**. Mice were anaesthetized with isoflurane and were placed in a stereotaxic frame using a mouse adaptor (David Kopf Instruments) with modified ear bars. Microdialysis probes were implanted in prefrontal cortex at the following coordinates according with mouse brain, relative to the bregma: AP +2.0 mm, ML +0.5 mm and DV –3.0 mm, with the tooth-bar also set at 0 mm. The active dialysis surface length of the membrane was 2 mm. The probe was secured in place with dental cement on the skull. Microdialysis experiments started 24 h after surgery. Ringer solution (125 mM NaCl, 2.5 mM KCl, 1.26 mM CaCl_2_, 1.18 mM MgCl_2_, 0.20 mM NaH_2_PO_4_) was perfused through the microdialysis probe at a flow at 1.0 μl/min using a high precision pump (CMA 400 syringe pump, CMA Sweden)^31^ The experiments were performed during the light period and the mice were tested in their home cage. D22 (D22, 1,1-diethyl-2,2-cyanine iodide, Sigma- Aldrich) (100 μM) was dissolved in Ringer solution and injected via probe. Deprenyl (10 mg/Kg i.p daily, Sigma-Aldrich) and L-DOPA/Benserazide (20 +12.5 mg/Kg i.p daily, Sigma-Aldrich) were dissolved in saline respectively and were administrated i.p. from postnatal day P25 until P38. After at least a 2 h equilibration period, the dialysates were collected every 30 min in a small Eppendorf tubes containing 11.7 μl acetic acid and stored at –69°C until HPLC analysis. The time course of extracellular levels of dopamine were performed in aVMAT2cKO and control LoxTAM mice from P23 to P26 during tamoxifene (100 mg kg^-1^ i.p.) injections. After at least a 2 h equilibration period, the dialysates were collected every 30 min for 2,5 h (5 samples for day). Dopamine levels were quantified by HPLC with electrochemical detection as described previously ^32^ with some modifications. Samples (20 µl) were injected onto an MD-150 column (3 µM, 3,2x150 mm Thermo Fisher Scientific) using a Thermo Scientific Dionex Ultimate 3000. Detection was performed at 32°C with an electrochemical detector (ECD-3000RS Thermo Fisher Scientific) setted at a potential of 250 mV against an Ag/AgCl reference electrode. The signal was analyzed using Cromeleon, software. The mobile phase was of 75 mM sodium dihydrogen phosphate monohydrate, 1,7 mM ottane sulphonic acid sodium, 100 µM trietilammine (TEA), 25 µM EDTA, 10% acetonitrile pH 3,00 with phosphoric acid. Flow rate was 0.5 ml/min. No experiments were discarded.

**In vivo electrophysiology**. Mice were anesthetized with isoflurane (Univentor, Malta, induction: 2%; maintenance: 1-1.5%), and placed in the stereotaxic apparatus (Kopf, Germany. Their body temperature was maintained at 36±1 °C using a heating pad (CMA 450 Temperature Controller, USA). The scalp was retracted and one burr hole was drilled above the right VTA (AP: -2.6 – -3.0 mm, L: 0.4–0.6 mm, V: 4.6–5.2) for the placement of a recording electrode. Single unit activity was recorded extracellularly by glass micropipettes filled with 2% pontamine sky blue dissolved in 0.5 M sodium acetate (impedance 3–6 MΩ). Signal was filtered (band-pass 500–5000 Hz), pre-amplified (DAM80, WPI, Germany), amplified and (Neurolog System, Digitimer, UK), displayed on a digital storage oscilloscope (OX 530, Metrix, USA). Experiments were sampled on- and off-line by a computer connected to CED Power 1401 laboratory interface (Cambridge Electronic Design, Cambridge, UK) running the Spike2 software (Cambridge Electronic Design). Single units were isolated and the spontaneous activity was recorded for 5m. Spontaneous firing rate, percentage of spikes in bursts and coefficient of variation (CV=standard deviation of interspike intervals divided by the mean interspike interval; a measure of firing regularity) were determined. Single units were isolated and identified according to previously described electrophysiological characteristics.^33,34,35^ We recorded VTA DA neurons only when criteria for identification were fulfilled (biphasic action potential of more than 1.1 ms duration).At the end of each experiment, the electrode placement was marked with an iontophoretic deposit of pontamine sky blue dye (−80 μA, continuous current for 5 min). Brains were then rapidly removed and fixed in 4% paraformaldehyde solution. The position of the electrodes was identified on serial sections (60 μm). No experiments were discarded.

**In vivo optogenetic experiments**. Mice were anaesthetized with isoflurane and were placed in a stereotaxic frame using a mouse adaptor (David Kopf Instruments) with modified ear bars. After ensuring sanitary and anesthetic depth as described previously, the skull was exposed in aseptic conditions, and small holes were drilled in the skull overlying the PFC and VTA. Optic fiber ferrules (200μm core diameter, Thorlabs) were implanted in the VTA at the following coordinates: AP - 3.4mm, ML +0.4mm, DV -4.4mm (from the bregma). ^36^ Microdialysis probes were implanted in prefrontal cortex at the following coordinates, relative to the bregma: AP +2.0 mm, ML +0.4 mm and DV –3.0 mm (adapted from ^37^), with the tooth-bar also set at 0 mm. The active dialysis surface length of the membrane was 2 mm. The probe and optic fiber ferrule were secured in place with dental cement on the skull. All photostimulation experiments were conducted unilaterally. Mice were injected with ketamine/xylazine prior to stimulation (80 mg per kg, intraperitoneal injection, then 40mg per kg after 100min and again 40mg per kg after 60 min). Light pulse trains were programmed and modulated using TTL input control software (Doric Lenses, Canada) and provided input to one blue-light laser (473nm, 20mW intensity, Shanghai Dream lasers). Phasic photostimulation firing was created with 25 pulses at 50Hz for 15ms for 20 minutes with a 1 minute periodicity. ^38^ The experiments were performed during the light period and the dialysates were collected every 20 min in a small Eppendorf tubes containing 7.8 μL acetic acid and stored at –69°C until HPLC analysis. No experiments were discarded.

**Morphological analysis of dendrites and spines.** Dendritic spine density and spine morphology was assessed as previously described. ^31^ For spine and dendrites analysis we used two fluorescent transgenic mice (aVMAT2cKO-Thy1EGFP and control LoxTAM-Thy1EGFP) obtained by the crossbreeding of Thy1EGFP 4 with aVMAT2cKO and VMAT2lox/lox, respectively. Confocal microscopy analysis was performed with a Leica confocal imaging system (TCS SP5) with a 40× (1.8 NA) or with 63x (2.8 NA) oil immersion objectives. Medial prefrontal cortex field between bregma coordinates 2.34mm to 1.7mm was analyzed. Brain sections (50 μm) were acquired every 0.4-μm thickness. Spines were imaged with confocal microscopy in postfixed slices from both control LoxTAM- Thy1EGFP and aVMAT2cKO-Thy1EGFP brains at P20, P28 and P40. Their morphology as well as the number of spine was measured using image J software, for 10-20 neurons per mouse. Spine density was expressed as the number of spines divided by dendritic length. Dendritic spine morphology were classed in 4 groups based on the maximal diameter of the spine head measured on maximal projections with Image J: filopodia spines < 0.25µm, stubby spines 0.25 to 0.60 (without neck), thin spines 0.25 to 0.60 µm and mushroom spines > 0.6 µm. ^31,39^ The percentage of each type of dendritic spine was then expressed by neuron and averaged for each mouse (10 to 20 neurons per group, ± 600 spines per group). No experiments were discarded.

**In vitro electrophysiology**. Transverse 400μm coronal sections of the prefrontal cortex were
prepared from P45-P60 old mice. Slices were transferred into a holding chamber where they were
maintained at 30°C in oxygenated (95% O2/5% CO2) artificial cerebro-spinal fluid (aCSF) composed of
(in mM): NaCl, 123; KCl, 2.5; Na2HPO4, 1; NaHCO3, 26.2; MgCl2, 2.5; CaCl2, 1 ; and glucose, 10. A
minimum of 1 hour was allowed for recovery prior to experimentation. When required, slices were
incubated 20 minutes in aCSF containing 0.5μM of the astrocytic marker sulforhodamine 101 and
then placed back in regular aCSF. For recordings, each slice was transferred to a recording chamber
mounted on the X--Y translation stage of an upright microscope (Zeiss Axioskop 2 FS+) and perfused
(2 ml/min) at 29°C with the extracellular solution (composition in mM : NaCl, 123; KCl, 2.5; Na2HPO4,
1; NaHCO3, 26.2; MgCl2, 1.3; CaCl2, 2.5 ; and glucose, 10). Field potentials were recorded with an
extracellular glass microelectrode placed in layer 5 of the prelimbic cortex and were evoked by
monopolar stimulation of layer II. Paired-pulse experiments consisted of 2 identical stimuli with
increasing interpulse intervals (50–250 ms, delivered at 0.066 Hz). Paired-pulse ratios were
generated by plotting the maximum slope of the second response as a percentage of the first (%
change). LTP was induced by high frequency tetanic stimulation consisting in six trains of 100 pulses
at 50Hz, with 10 seconds inter-train interval. Test shock intensity was set to one third of the maximal
fEPSP slope. Recordings of neuronal excitability was performed by somatic whole cell recordings in
the current clamp mode using a K-gluconate based solution (in mM: K-gluconate,120; KCl, 20; HEPES,
10, EGTA, 1; MgCl2, 1; CaCl2, 0.1) and consisted in injecting depolarizing pulse of increasing
intensities (0-200pA; 25pA increments). Negative current pulses were also applied in order to
calculate passive properties; i.e. resting potential, input resistance, membrane time constants and
capacitance. Whole-cell patch-clamp recordings of miniature EPSCs isolated by 50μm picrotoxin and
1μM tetrodotoxin were obtained. Pipette resistance was 3–6 MΩ. Series resistances were
uncompensated and cells with Ra > 20 MΩ or cells for which a >20% change in Ra occurred during
the experiment were excluded from analyses. mEPSCs were isolated using ElphyTM (Biologic UNICCNRS, Gif sur Yvette, France) with a detection threshold of 5 pA. Cells with an access resistance > 25 MΩ at resting potential were excluded from analyses as well as any cell for which a change >20 % in those parameters occurred during the course of the experiment.

**Behavioral Protocols.**

**T-maze alternation task**: T-maze was used to evaluate working memory. Briefly, black plastic T-maze (stem, 38 x 10 cm; arms, 38 x 10 cm; walls, 12.7 cm high). Sliding doors separated the first 12.7 cm of the stem as the starting compartment, and the arms from the stem from the intersection. The end of each arm contained a small blue plastic cup (1.5 cm in diameter) into which a food reward was placed. A variety of fixed extra-maze clues surrounded the apparatus. Mice were kept on a maintenance diet throughout the course of all T-maze experiments and they lost 10 – 15 % of their normal body weight. In the habituation sessions, which lasted 4 days, mice were placed in the starting arm of the maze and allowed to explore it freely for 10 min, with a milk drop provided as a reward at the end of both side (goal) arms. After the habituation phase, mice were trained for 15 trials per day over 12 days. In the first trial, the reward was placed in both goal arms. Over the subsequent 15 trials, only one of the goal arms was baited; if the mouse had successfully found the bait on the previous trial, the arm opposite to the one it had just entered was baited; if it had gone to the empty arm on the last trial, the reward was left in the same place. Thus, animals had to learn to alternate between both arms to find the reward. T-maze delayed non-match-to-place (DNMTP): The delayed alternation test was conducted in a black plastic T-maze (stem, 38 x 10 cm; arms, 38 x 10 cm; walls, 12.7 cm high). Sliding doors separated the first 12.7 cm of the stem as the starting compartment, and the arms from the stem from the intersection. The end of each arm contained a small blue plastic cup (1.5 cm in diameter) into which a food reward was placed. A variety of fixed extra-maze clues surrounded the apparatus. Mice were kept on a maintenance diet throughout the course of all T-maze experiments and they lost 10 – 15 % of their normal body weight. After 2 days of dieting, animals were subjected to 4 adaptation/shaping sessions (1 session per day for 4 consecutive days), during which they were allowed to explore the T-maze and obtain rewards (milk drop). In each adaptation session, mice were subjected to 1 free choice trail (both arms were baited) and 10 forced alternation trials (5 left and 5 right) in which mice were forced (by blocking access to the previously visited arm) to visit one arm each time, eat the food reward, and return to the starting compartment. Mice were confined to the starting compartment for about 5 sec between trials. Mice were returned to their home cages after their daily training.

Actual training sessions commenced 1 day after the last adaptation session. In the training sessions, mice were subjected to 11 continuous trials (1 free choice trial followed by 10 test trials) with 5 sec delay between trials for 5 consecutive days. During the 10 test trials, the food reward was always located in the arm not visited in the previous trial. A correct choice was made if the mouse entered the previously unvisited arm. The baited arm remained the same until visited, even if the mouse chose the incorrect arm repeatedly. After completion of the 5-day training sessions, mice were trained using the same training protocol as described above until they reached the testing criteria that is the accuracy of arm choosing is equal to or above 70% (7 of 10 test trials) for 2 consecutive days. One day after reaching the testing criteria, animals were subjected to a memory test consisting of 1 free choice trial followed by 10 5-sec delay trials and 10 20-sec delay trials in semi-random order. Working memory errors (5-sec errors and 20-sec errors) were recorded. Mice that were not interested in the reward mechanism were excluded from the behavior tests.

**Odor discrimination**: The odor discrimination task used was exactly as described previously. ^40^ Briefly, mice were trained to dig to retrieve a food reward (milk pellet, Sigma) in digging medium scented with various odor. Mice were trained to discriminate between two odors until they reached criterion of 6 consecutively correct trials (Day 1, simple discrimination). The following day mice were tested using the same two odors (Day 2, repetition), after criterion was reached the odor association was switched and the previously unbaited odor was now baited (Day 2 rev, reversal). Number of trials to reach the criterion was recorded. Mice that were not interested in the reward mechanism were excluded from the behavior tests.

**Statistical Analysis**. Samples size (n) are indicated in the results section. Sample sizes were based on pilot experiments and previous works. Animals were characterized (depending on the experiment) and randomly allocated to groups. Investigators were blinded to the groups during the experiments, where applicable. All analyses were performed using GraphPad Prism 6.0 software. Then, a one- way ANOVA was performed followed by Bonferroni’s post hoc tests or Tukey post hoc test. For two sample comparisons, unpaired t-test was used. For behaviour procedures and in vitro electrophysiology, parametric tests were used: two-way ANOVA with repeated measures followed by Bonferroni’s post hoc tests or Tukey post hoc test. All data had similar variances and are presented as mean ± SEM. Statistical significant was considered at the p<0.05 level.

**Supplementary references**

1. Hirrlinger PG, Scheller A, Braun C, Hirrlinger J, Kirchhoff F. Temporal control of gene recombination in astrocytes by transgenic expression of the tamoxifen-inducible DNA recombinase variant CreERT2. Glia 2006; 54(1): 11-20.

2. Narboux-Neme N, Sagne C, Doly S, Diaz SL, Martin CBP, Angenard G et al. Severe Serotonin Depletion after Conditional Deletion of the Vesicular Monoamine Transporter 2 Gene in Serotonin Neurons: Neural and Behavioral Consequences. Neuropsychopharmacology 2011; 36(12): 2538-2550.

3. Fon EA, Pothos EN, Sun BC, Killeen N, Sulzer D, Edwards RH. Vesicular transport regulates monoamine storage and release but is not essential for amphetamine action. Neuron 1997; 19(6): 1271-1283.

4. Feng GP, Mellor RH, Bernstein M, Keller-Peck C, Nguyen QT, Wallace M et al. Imaging neuronal subsets in transgenic mice expressing multiple spectral variants of GFP. Neuron 2000; 28(1): 41-51.

5. Srinivas S, Watanabe T, Lin CS, William CM, Tanabe Y, Jessell TM et al. Cre reporter strains produced by targeted insertion of EYFP and ECFP into the ROSA26 locus. BMC developmental biology 2001; 1: 4.

6. Lioy DT, Garg SK, Monaghan CE, Raber J, Foust KD, Kaspar BK et al. A role for glia in the progression of Rett's syndrome. Nature 2011; 475(7357): 497-U490.

7. Bezzi P, Gundersen V, Galbete JL, Seifert G, Steinhauser C, Pilati E et al. Astrocytes contain a vesicular compartment that is competent for regulated exocytosis of glutamate. Nature Neuroscience 2004; 7(6): 613-620.

8. Marchaland J, Cali C, Voglmaier SM, Li HY, Regazzi R, Edwards RH et al. Fast subplasma membrane Ca2+ transients control exo-endocytosis of synaptic-like microvesicles in astrocytes. Journal of Neuroscience 2008; 28(37): 9122-9132.

9. Buscemi, L., Ginet, V., Lopatar, J., Montana, V., Pucci, L., Spagnuolo, P., Zehnder, T., Grubisci, V., Truttman, A., Sala, C., Hirt, L., Parpura, V., Puyal, J., Bezzi, P. (2016). Homer 1 scaffold proteins govern Ca2+ dynamics in normal and reactive astrocytes. Cerebral Cortex, 1-20.

10. Hirrlinger PG, Scheller A, Braun C, Quintela-Schneider M, Fuss B, Hirrlinger J et al. Expression of reef coral fluorescent proteins in the central nervous system of transgenic mice. Molecular and Cellular Neuroscience 2005; 30(3): 291-303.

11. Cahoy JD, Emery B, Kaushal A, Foo LC, Zamanian JL, Christopherson KS et al. A transcriptome database for astrocytes, neurons, and oligodendrocytes: A new resource for understanding brain development and function. Journal of Neuroscience 2008; 28(1): 264-278.

12. Colin A, Faideau M, Dufour N, Auregan G, Hassig R, Andrieu T et al. Engineered Lentiviral Vector Targeting Astrocytes In Vivo. Glia 2009; 57(6): 667-679.

13. Sohal VS, Zhang F, Yizhar O, Deisseroth K. Parvalbumin neurons and gamma rhythms enhance cortical circuit performance. Nature 2009; 459(7247): 698-702.

14. Zhang SL, Qi J, Li XP, Wang HL, Britt JP, Hoffman AF et al. Dopaminergic and glutamatergic microdomains in a subset of rodent mesoaccumbens axons. Nature Neuroscience 2015; 18(3): 386-392

15. Ravizza T, Vezzani A. Status epilepticus induces time-dependent neuronal and astrocytic expression of interleukin-1 receptor type I in the rat limbic system. Neuroscience 2006; 137(1): 301-308.

16. Kanazawa H, Ohsawa K, Sasaki Y, Kohsaka S, Imai Y. Macrophage/microglia-specific protein Iba1 enhances membrane ruffling and Rac activation via phospholipase C-gamma-dependent pathway. Journal of Biological Chemistry 2002; 277(22): 20026-20032.

17. Zhang L, Yu J, Park BH, Kinzler KW, Vogelstein B. Role of BAX in the apoptotic response to anticancer agents. Science 2000; 290(5493): 989-992.

18. Wang YM, Gainetdinov RR, Fumagalli F, Xu F, Jones SR, Bock CB et al. Knockout of the vesicular monoamine transporter 2 gene results in neonatal death and supersensitivity to cocaine and amphetamine. Neuron 1997; 19(6): 1285-1296.

19. Schubert V, Bouvier D, Volterra A. SNARE Protein Expression in Synaptic Terminals and Astrocytes in the Adult Hippocampus: A Comparative Analysis. Glia 2011; 59(10): 1472-1488.

20. Knott GW, Holtmaat A, Wilbrecht L, Welker E, Svoboda K. Spine growth precedes synapse formation in the adult neocortex in vivo. Nature Neuroscience 2006; 9(9): 1117-1124.

21. Magavi SS, Leavitt BR, Macklis JD. Induction of neurogenesis in the neocortex of adult mice. Nature 2000; 405(6789): 951-955.

22. Cui M, Aras R, Christian WV, Rappold PM, Hatwar M, Panza J et al. The organic cation transporter- 3 is a pivotal modulator of neurodegeneration in the nigrostriatal dopaminergic pathway. Proceedings of the National Academy of Sciences of the United States of America 2009; 106(19): 8043-8048.

23. Fenelon K, Mukai J, Xu B, Hsu PK, Drew LJ, Karayiorgou M et al. Deficiency of Dgcr8, a gene disrupted by the 22q11.2 microdeletion, results in altered short-term plasticity in the prefrontal cortex. Proceedings of the National Academy of Sciences of the United States of America 2011; 108(11): 4447-4452.

24. Monai H, Ohkura M, Tanaka M, Oe Y, Konno A, Hirai H et al. Calcium imaging reveals glial involvement in transcranial direct current stimulation-induced plasticity in mouse brain. Nature Communications 2016; 7: 10.

25. Jo S, Yarishkin O, Hwang YJ, Chun YE, Park M, Woo DH et al. GABA from reactive astrocytes impairs memory in mouse models of Alzheimer's disease. Nature Medicine 2014; 20(8): 886-896.

26. Calì, C., Baghabra, J., Boges, D.J., Holst, G.R., Kreshuk, A., Hamprecht, F.A., Srinivasan, M., Lehväslaiho, H., and Magistretti, P.J. (2016). Three-dimensional immersive virtual reality for studying cellular compartments in 3D models from EM preparations of neural tissues: 3D Virtual reality for neural tissue. J. Comp. Neurol. 524, 23–38.

27. Jorstad, A., Nigro, B., Cali, C., Wawrzyniak, M., Fua, P., and Knott, G. (2015). NeuroMorph: A Toolset for the Morphometric Analysis and Visualization of 3D Models Derived from Electron Microscopy Image Stacks. Neuroinformatics 13, 83–92.

28. Meira LB, Moroski-Erkul CA, Green SL, Calvo JA, Bronson RT, Shah D et al. Aag-initiated base excision repair drives alkylation-induced retinal degeneration in mice. Proceedings of the National Academy of Sciences of the United States of America 2009; 106(3): 888-893.

29. Bergersen LH, Storm-Mathisen J, Gundersen V. Immunogold quantification of amino acids and proteins in complex subcellular compartments. Nature Protocols 2008; 3(1): 144-152.

30. Dunand M, Gubian D, Stauffer M, Abid K, Grouzmann E. High-Throughput and Sensitive Quantitation of Plasma Catecholamines by Ultraperformance Liquid Chromatography-Tandem Mass Spectrometry Using a Solid Phase Microwell Extraction Plate. Analytical Chemistry 2013; 85(7): 3539- 3544.

31. Sultan S, Li LY, Moss J, Petrelli F, Casse F, Gebara E et al. Synaptic Integration of Adult-Born Hippocampal Neurons Is Locally Controlled by Astrocytes. Neuron 2015; 88(5): 16.

32. Nirenberg MJ, Chan J, Liu YJ, Edwards RH, Pickel VM. Ultrastructural localization of the vesicular monoamine transporter-2 in midbrain dopaminergic neurons: Potential sites for somatodendritic storage and release of dopamine. Journal of Neuroscience 1996; 16(13): 4135-4145.

33. Grace, A.A., and Bunney, B.S. (1983). Intracellular and extracellular electrophysiology of nigral dopaminergic-neurons .2. action-potential generating mechanisms and morphological correlates. Neuroscience 10, 317-31.

34. Grace, A.A., and Bunney, B.S. (1984). The control of firing pattern in nigral dopamine neurons - burst firing. Journal of Neuroscience 4, 2877-2890.

35. Ungless, M.A., and Grace, A.A. (2012). Are you or aren't you? Challenges associated with physiologically identifying dopamine neurons. Trends in Neurosciences 35, 422-430.

36. Tan KR, Yvon C, Turiault M, Mirzabekov JJ, Doehner J, Labouebe G et al. GABA Neurons of the VTA Drive Conditioned Place Aversion. Neuron 2012; 73(6): 1173-1183.

37. Tritsch NX, Sabatini BL. Dopaminergic Modulation of Synaptic Transmission in Cortex and Striatum. Neuron 2012; 76(1): 33-50.

38. Tsai HC, Zhang F, Adamantidis A, Stuber GD, Bonci A, de Lecea L et al. Phasic Firing in Dopaminergic Neurons Is Sufficient for Behavioral Conditioning. Science 2009; 324(5930): 1080-1084.

39. Crimins JL, Rocher AB, Peters A, Shultz P, Lewis J, Luebke JI. Homeostatic responses by surviving cortical pyramidal cells in neurodegenerative tauopathy. Acta Neuropathologica 2011; 122(5): 551- 564.

40. Colacicco G, Welzl H, Lipp HP, Wurbel H. Attentional set-shifting in mice: modification of a rat paradigm, and evidence for strain-dependent variation. Behavioural Brain Research 2002; 132(1): 95- 102.

odr
