# Supplementary figures and images for: Dysfunction of homeostatic control of dopamine by astrocytes in the developing prefrontal cortex leads to cognitive impairments
Source: Mol Psychiatry. 2018 Aug 20;25(4):732–49. doi: 10.1038/s41380-018-0226-y (PMC7156348; doi:10.1038/s41380-018-0226-y)

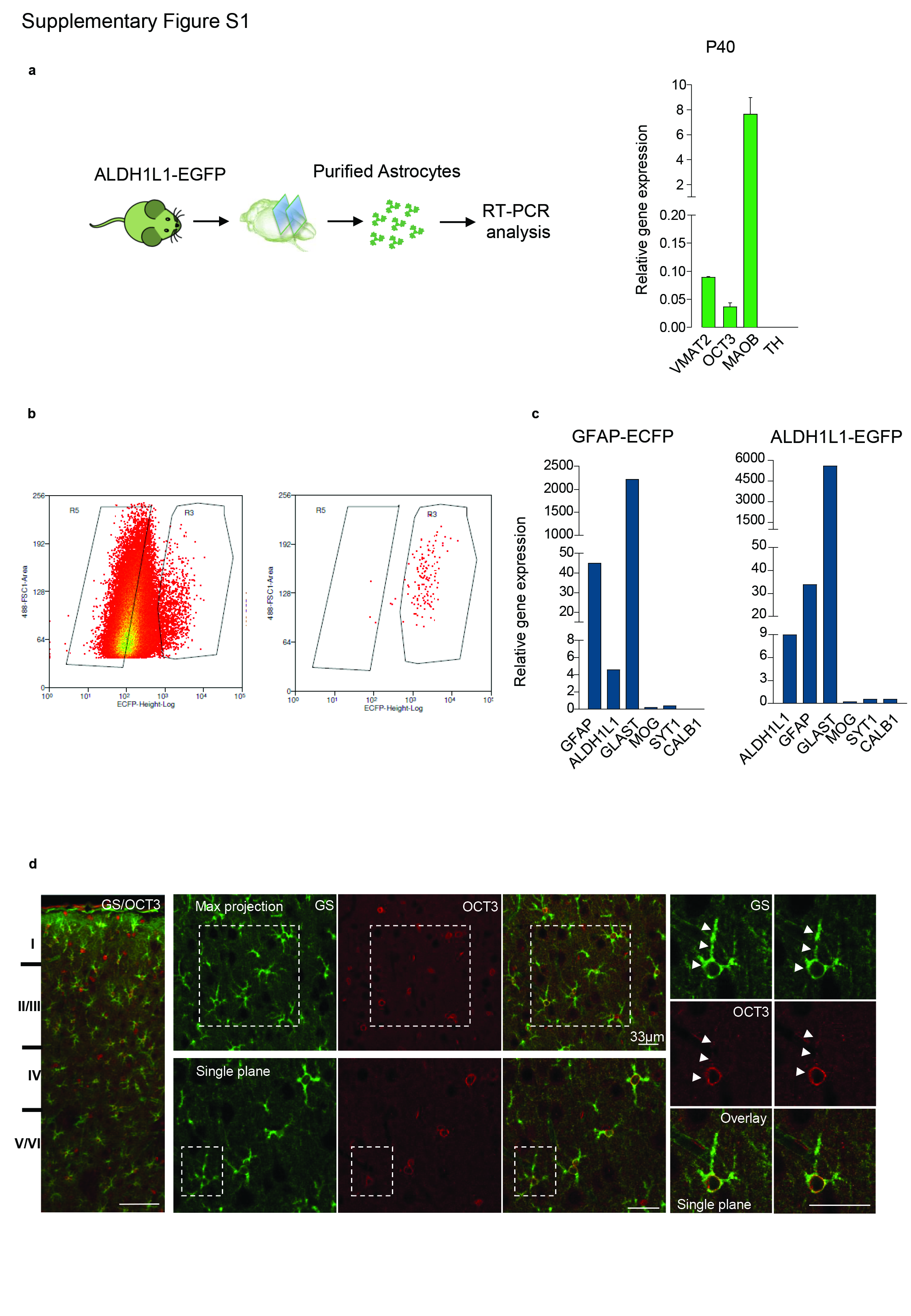

Supplement: Supplementary file 7 — Supplementary Fig S1a-d [file 41380_2018_226_MOESM7_ESM.tif]

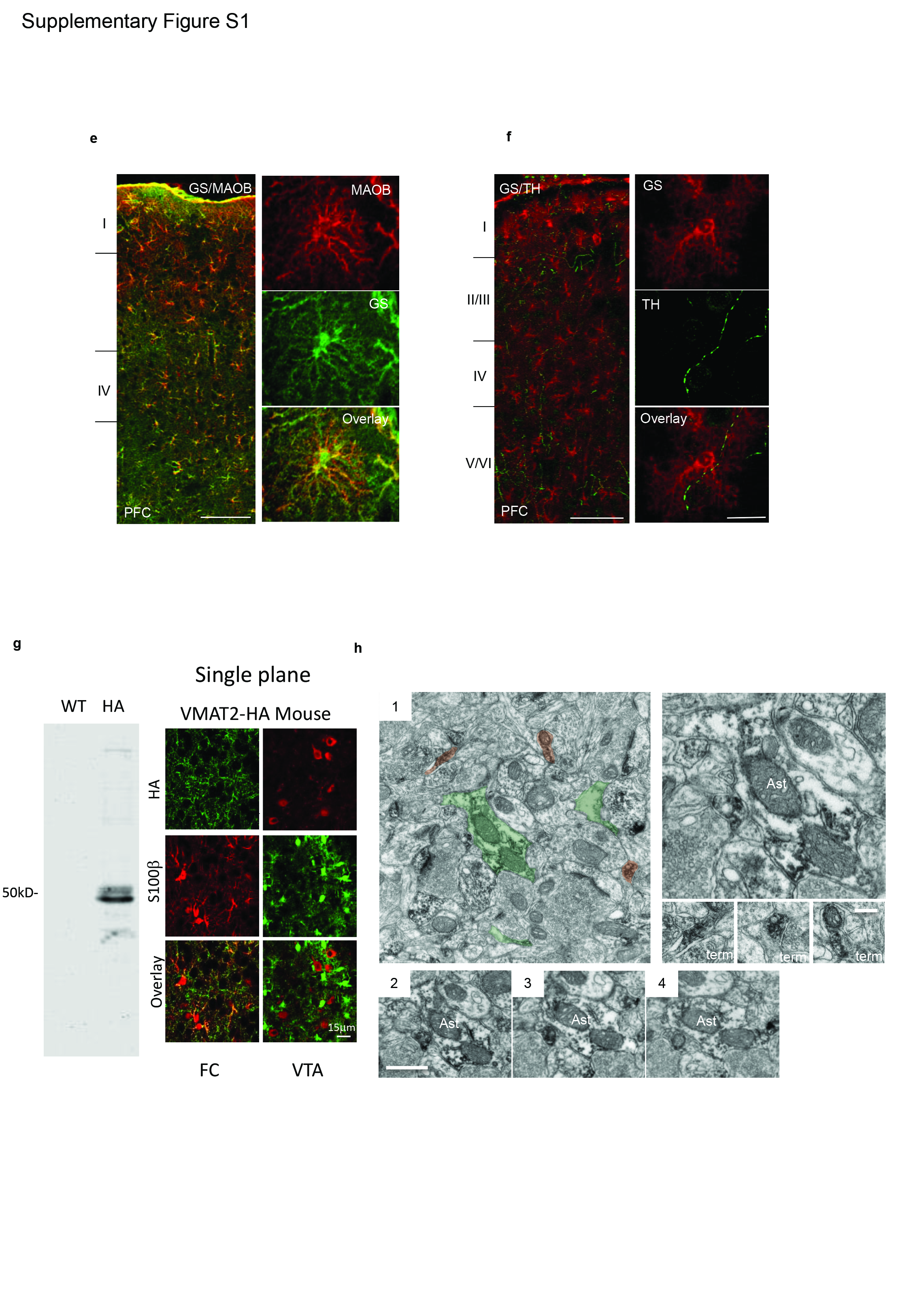

Supplement: Supplementary file 8 — Supplementary Fig S1e-h [file 41380_2018_226_MOESM8_ESM.tif]

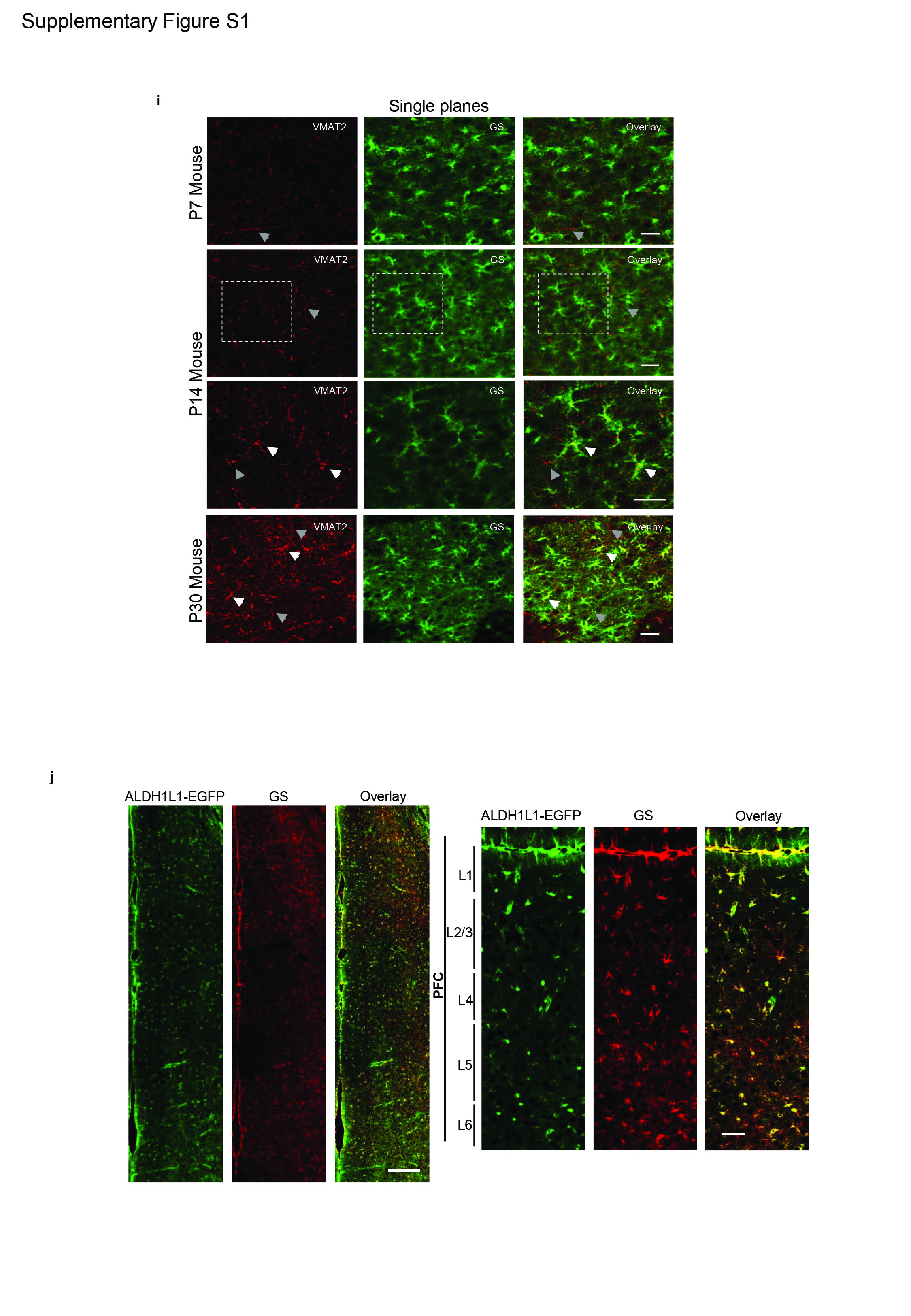

Supplement: Supplementary file 9 — Supplementary Fig S1i-j [file 41380_2018_226_MOESM9_ESM.tif]

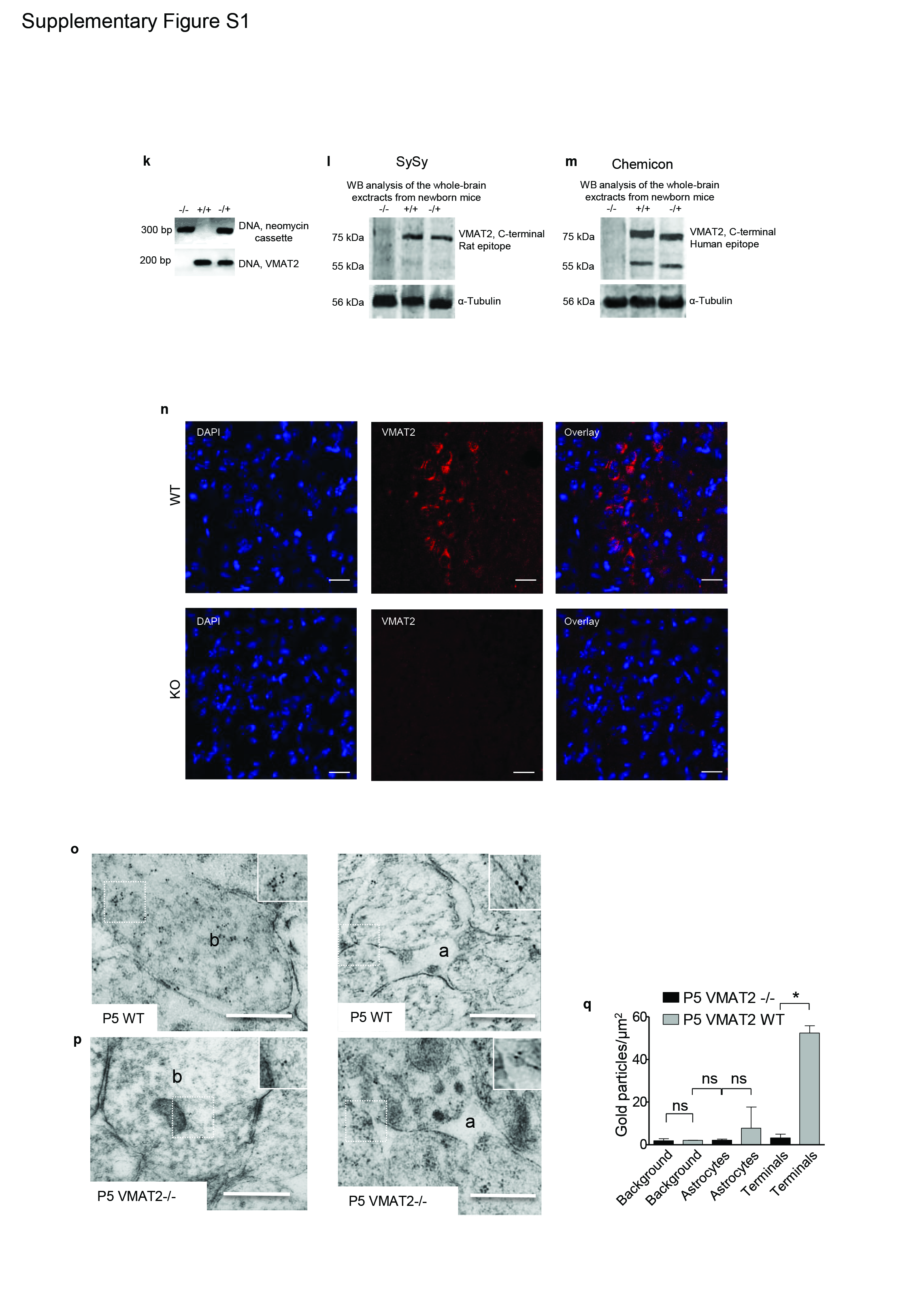

Supplement: Supplementary file 10 — Supplementary Fig S1k-q [file 41380_2018_226_MOESM10_ESM.tif]

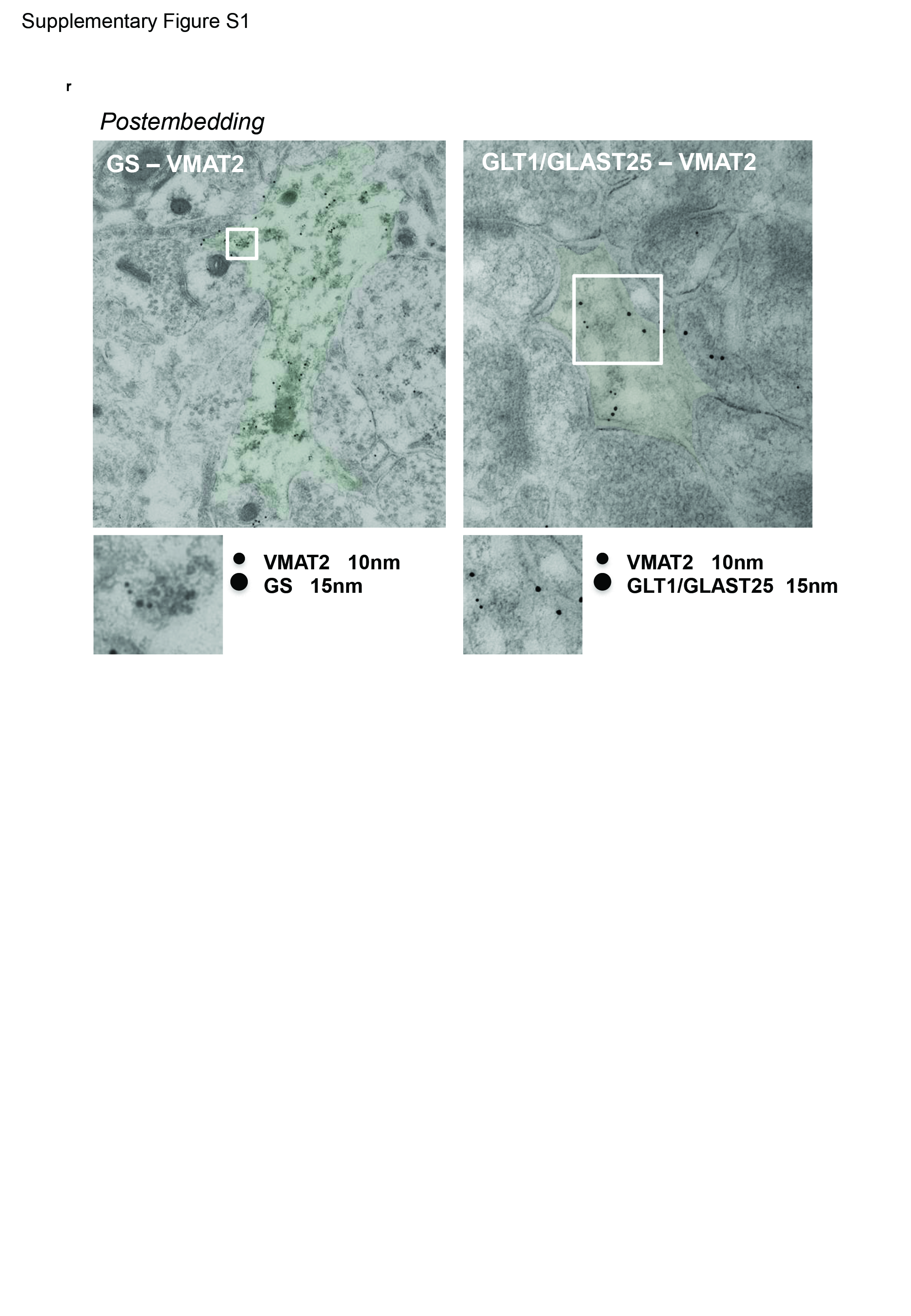

Supplement: Supplementary file 11 — Supplementary Fig S1r [file 41380_2018_226_MOESM11_ESM.tif]

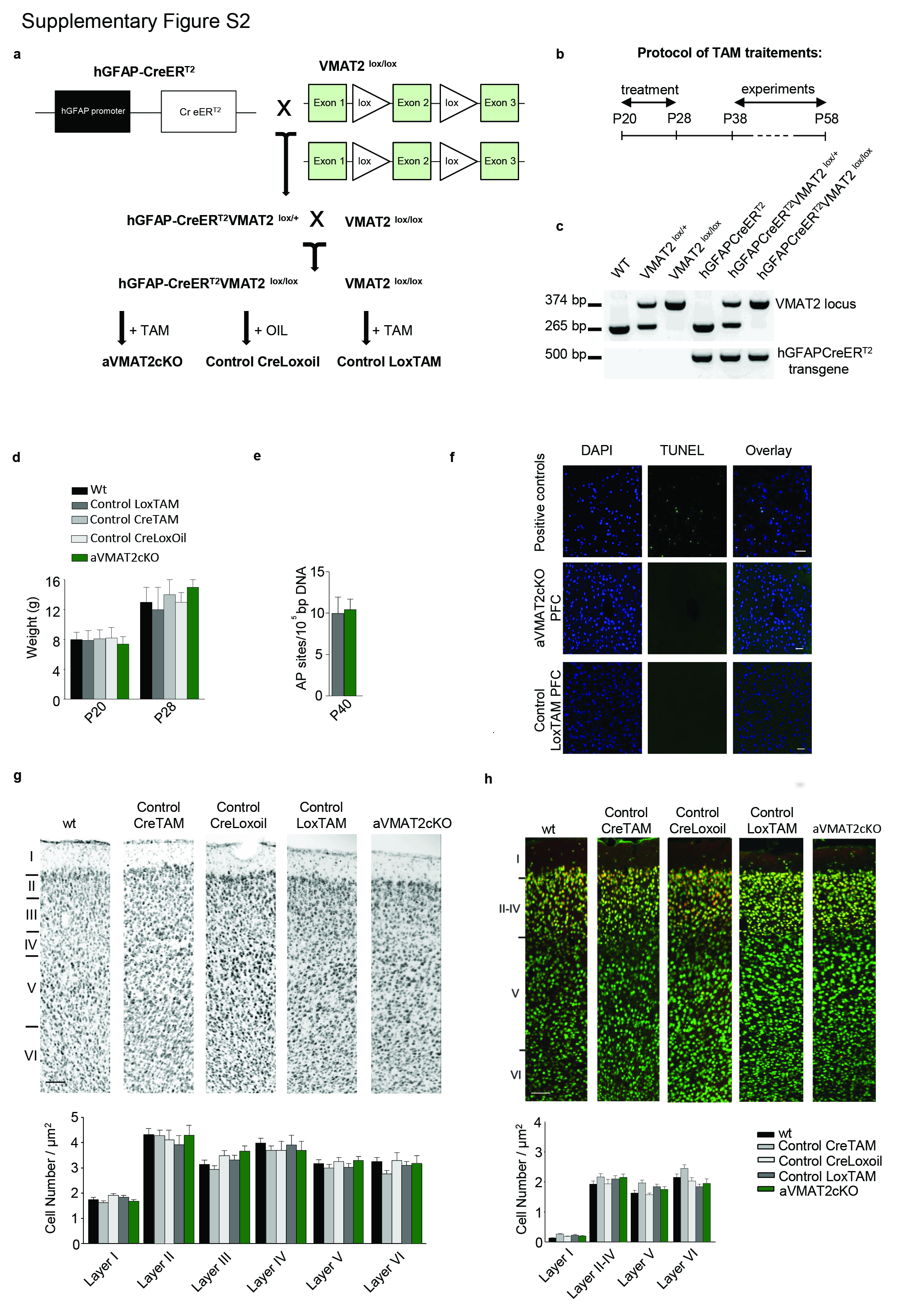

Supplement: Supplementary file 12 — Supplementary Fig S2a-h [file 41380_2018_226_MOESM12_ESM.tif]

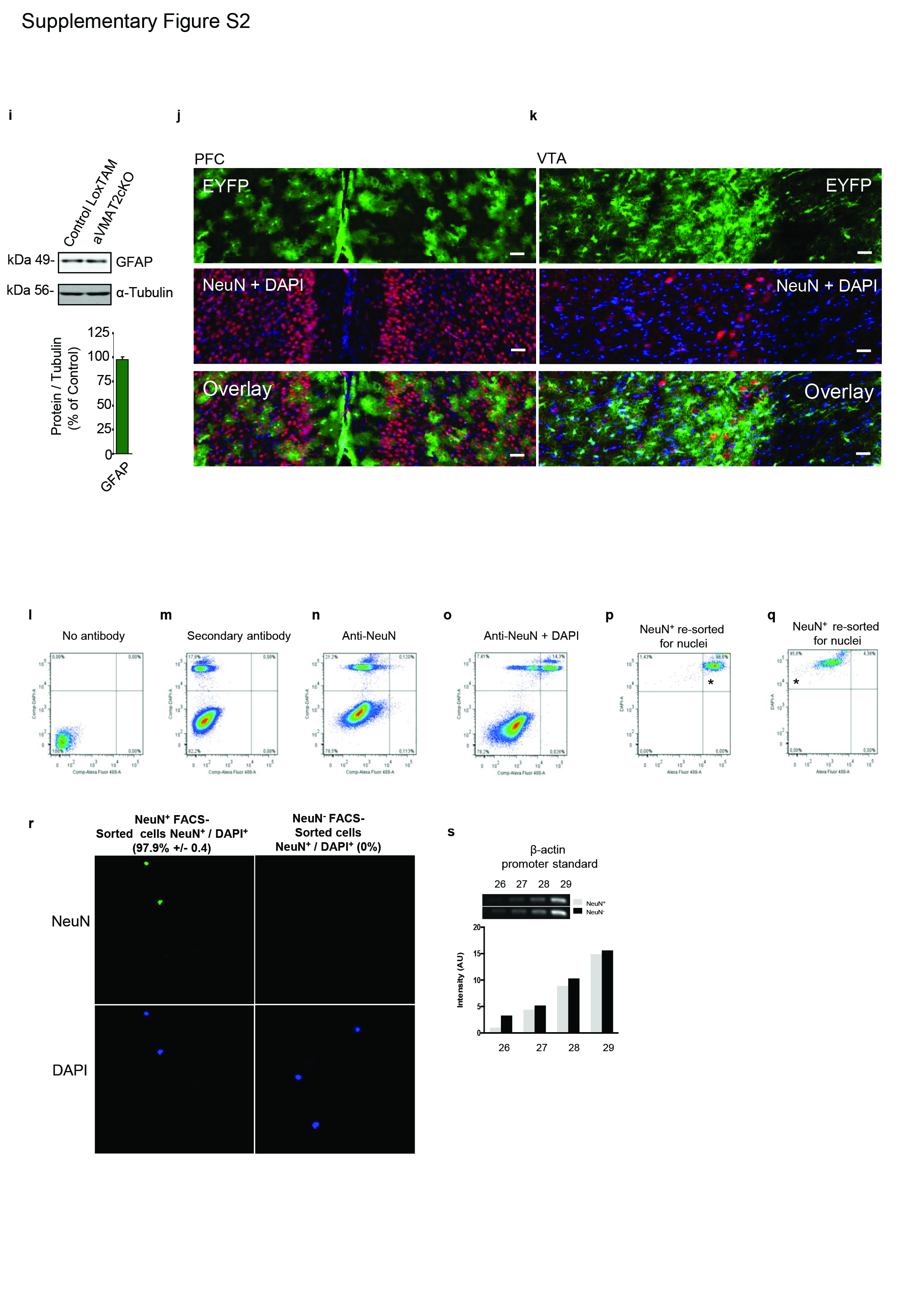

Supplement: Supplementary file 13 — Supplementary Fig S2i-s [file 41380_2018_226_MOESM13_ESM.tif]

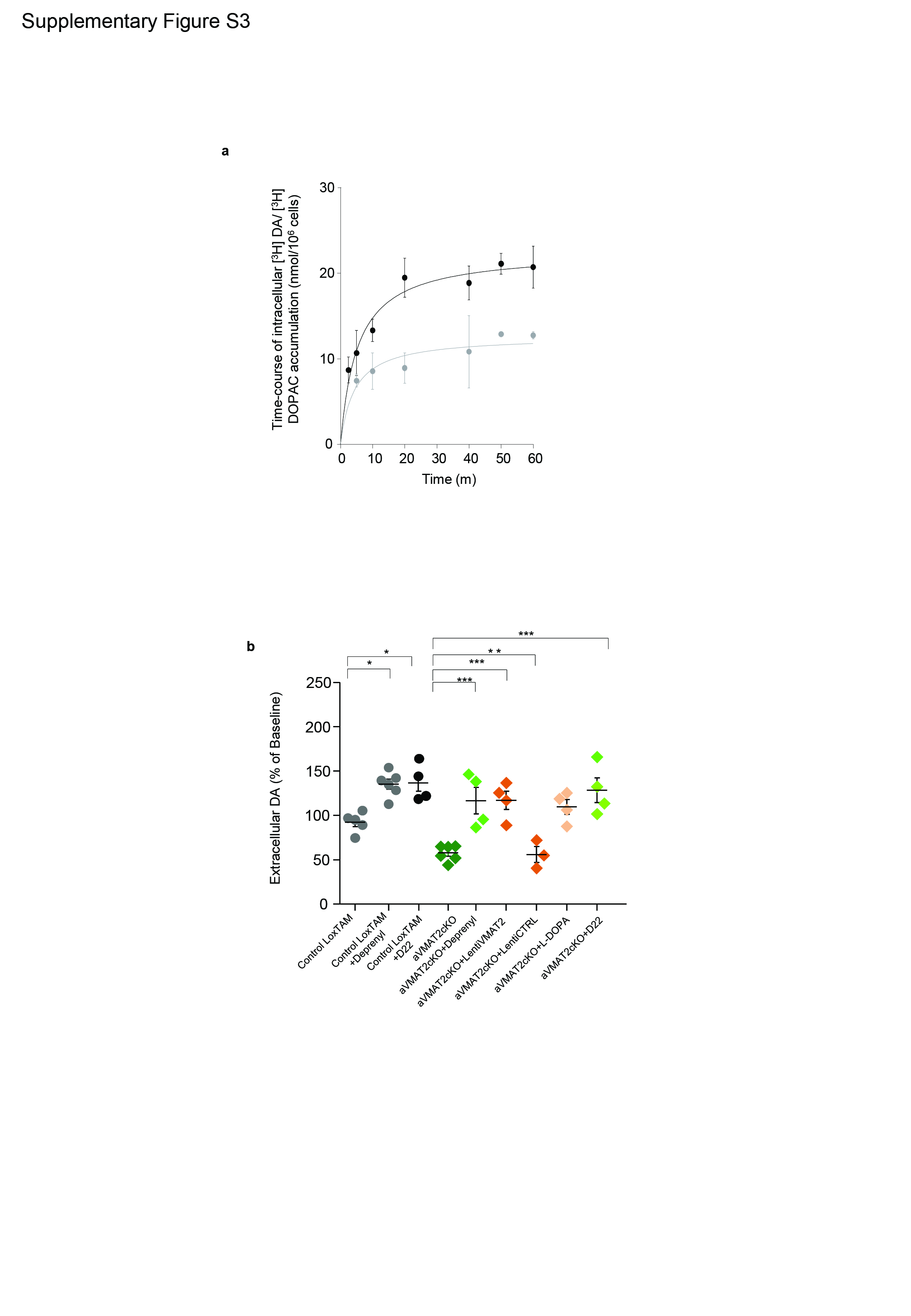

Supplement: Supplementary file 14 — Supplementary Fig S3a-b [file 41380_2018_226_MOESM14_ESM.tif]

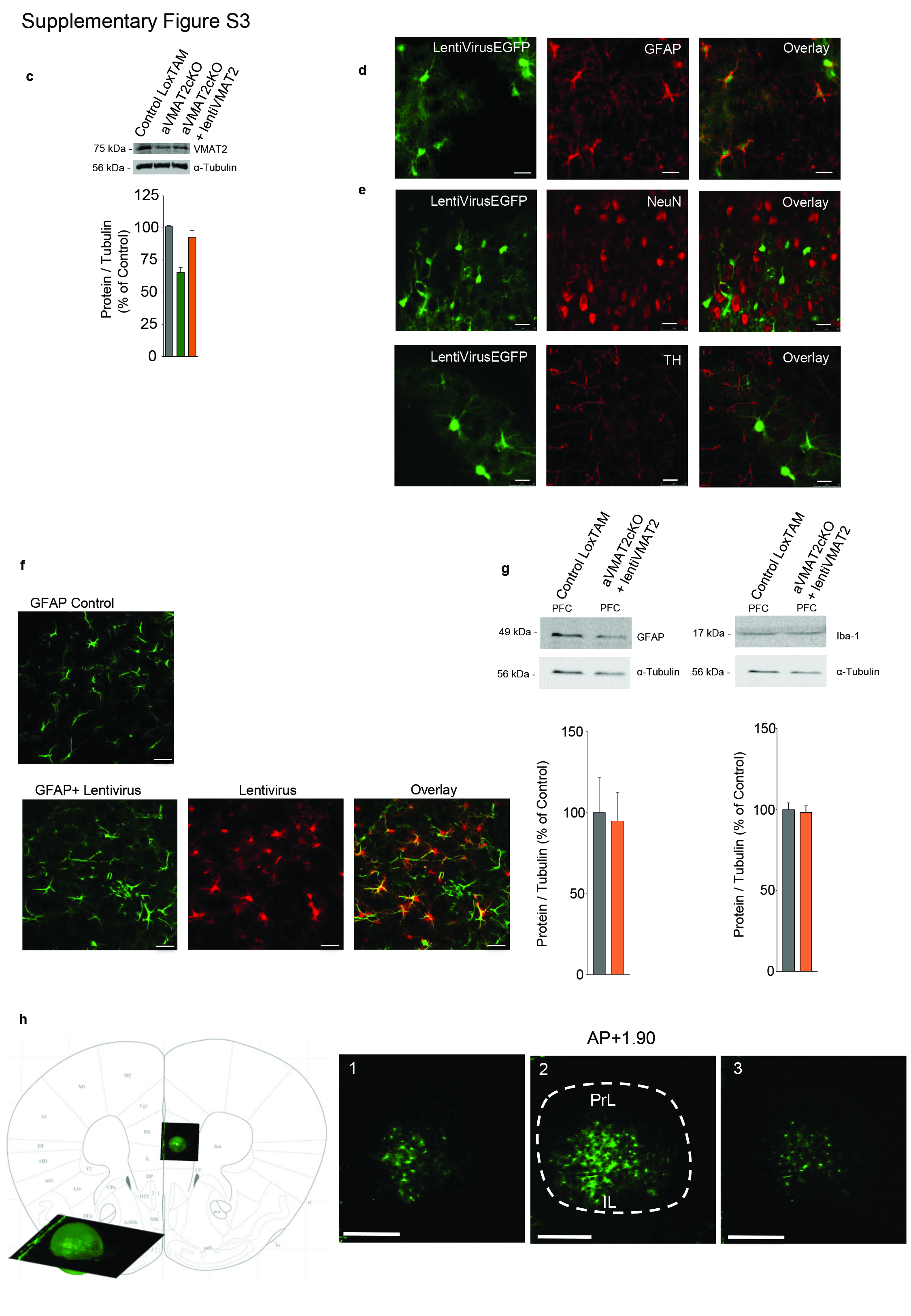

Supplement: Supplementary file 15 — Supplementary Fig S3c-h [file 41380_2018_226_MOESM15_ESM.tif]

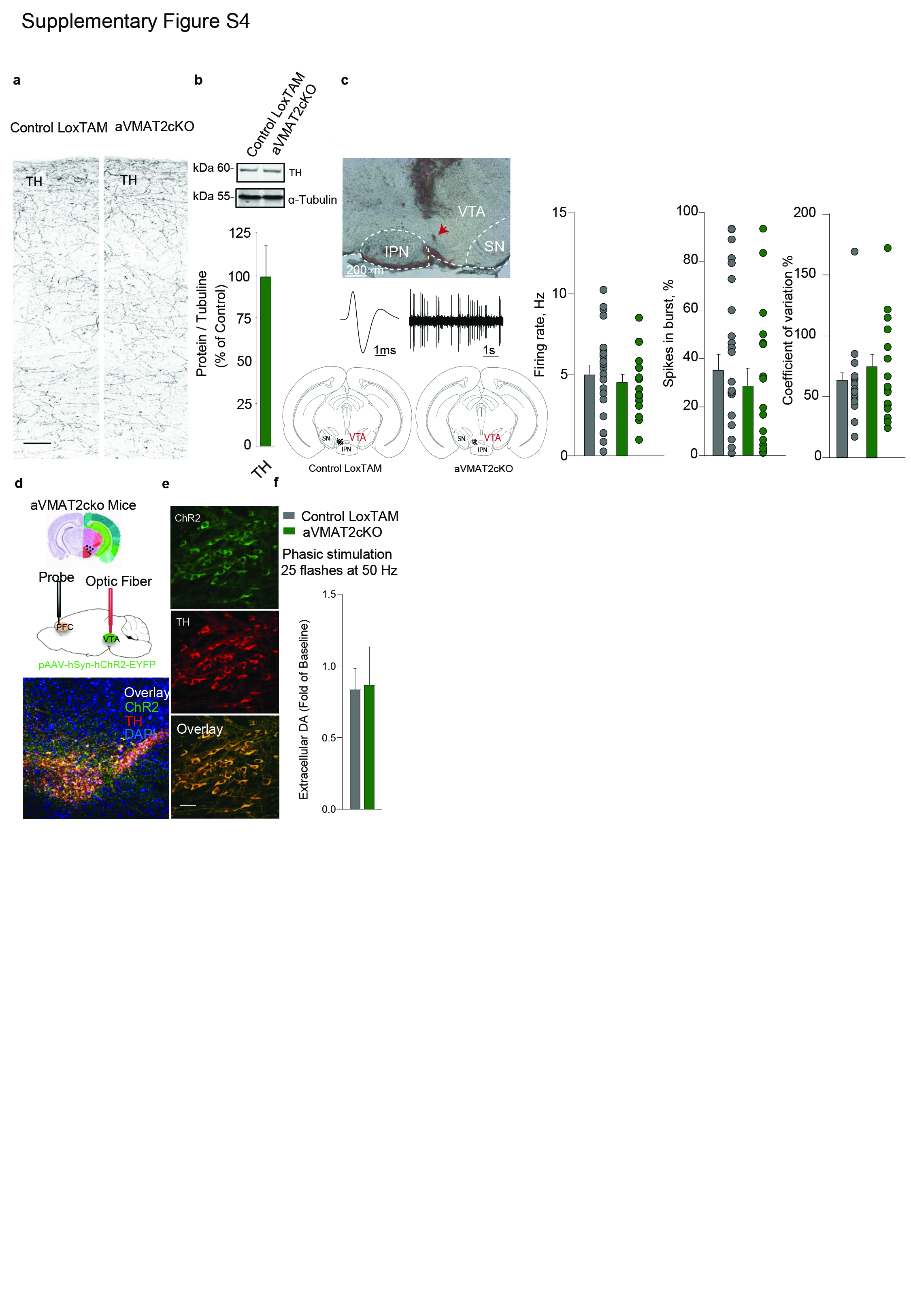

Supplement: Supplementary file 16 — Supplementary Fig S4a-f [file 41380_2018_226_MOESM16_ESM.tif]

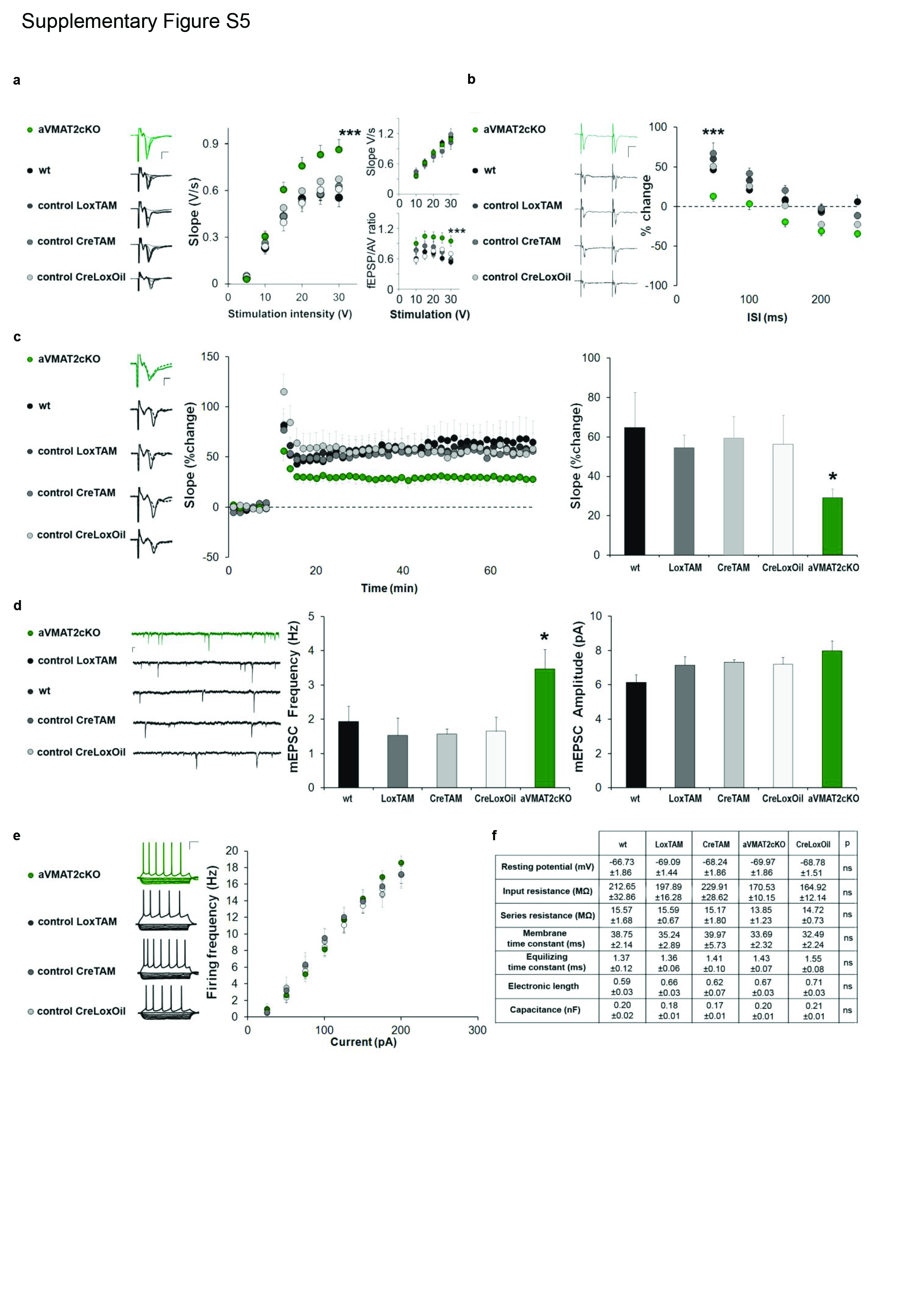

Supplement: Supplementary file 17 — Supplementary Fig S5a-f [file 41380_2018_226_MOESM17_ESM.tif]

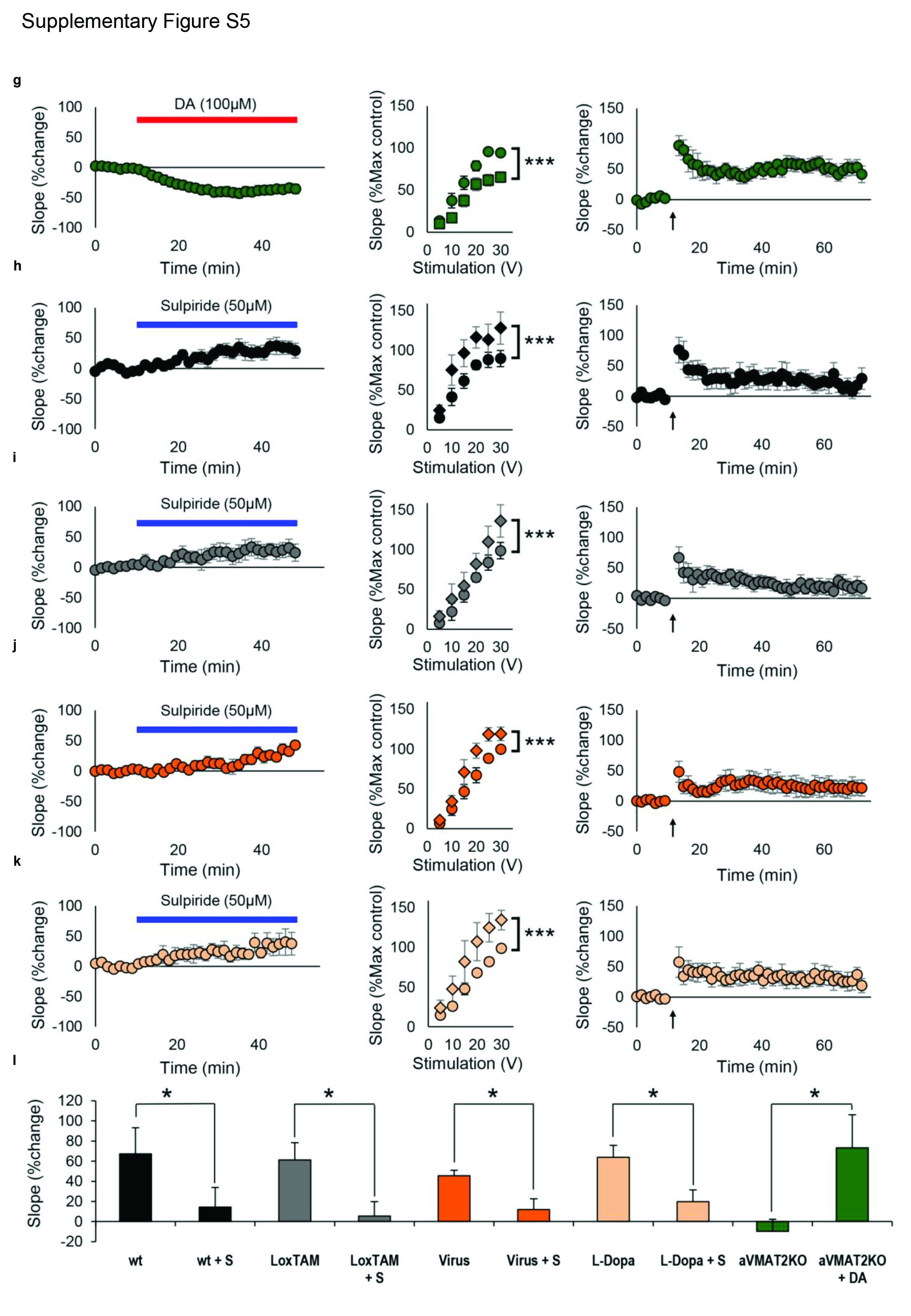

Supplement: Supplementary file 18 — Supplementary Fig S5g-l [file 41380_2018_226_MOESM18_ESM.tif]

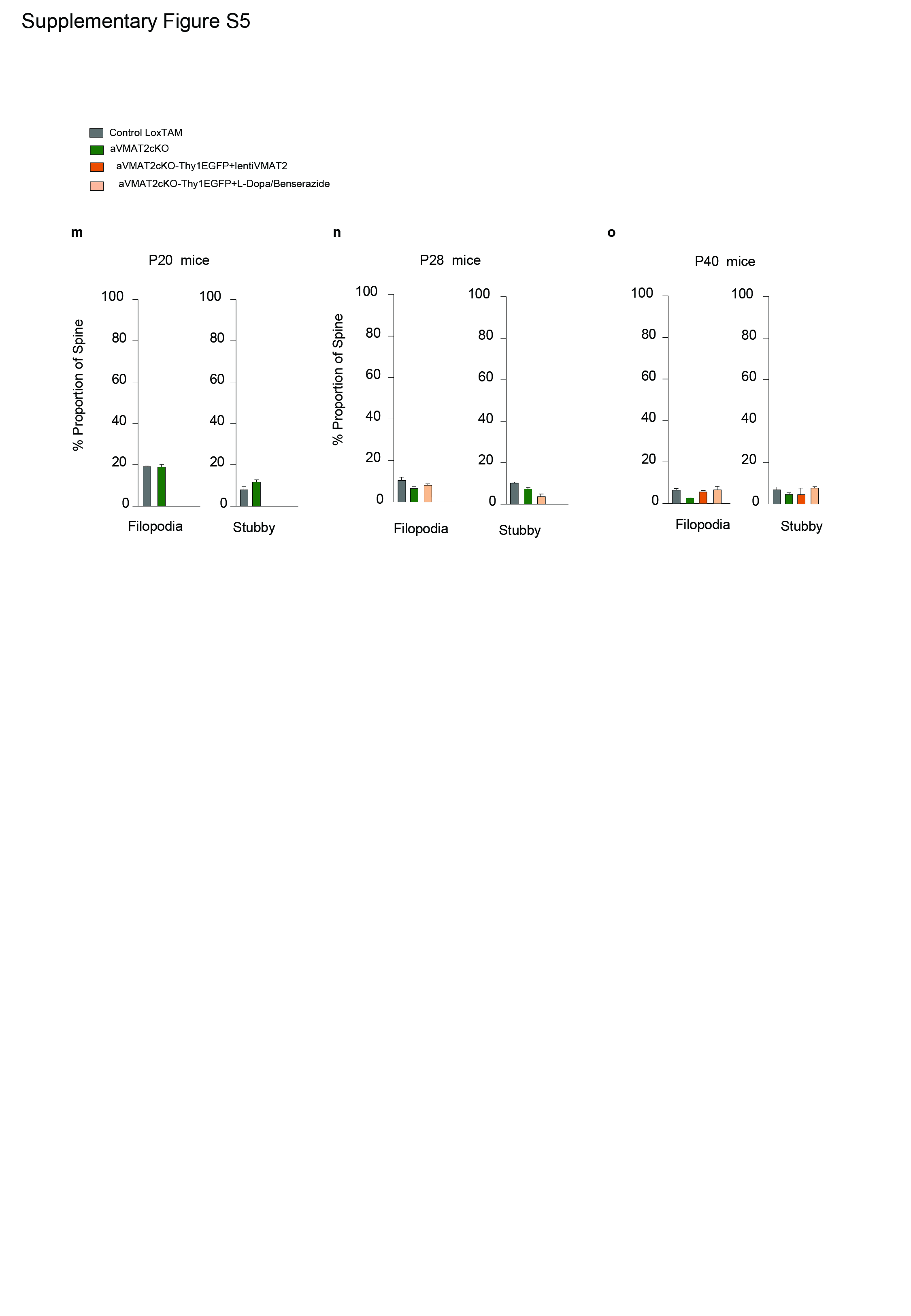

Supplement: Supplementary file 19 — Supplementary Fig S3m-o [file 41380_2018_226_MOESM19_ESM.tif]
